# Lymphotoxin beta receptor^-/-^ mice display altered B- and T-cell subpopulations in the bone marrow and peritoneal cavity after *Toxoplasma gondii* infection

**DOI:** 10.1128/iai.00408-25

**Published:** 2025-09-09

**Authors:** Marcel Helle, Ursula R. Sorg, Johannes Ptok, Rachel E. Thomas, Katharina Pracht, Patrick Petzsch, Alain de Bruin, Hans-Martin Jäck, Karl Köhrer, Daniel Degrandi, Klaus Pfeffer

**Affiliations:** 1 Institute of Medical Microbiology and Hospital Hygiene, Heinrich Heine University 9170, Düsseldorf, Germany; 2 Institute of Virology, Heinrich Heine University 9170, Düsseldorf, Germany; 3 Department Biomolecular Health Sciences, Faculty of Veterinary Medicine, Utrecht University 8125, Utrecht, the Netherlands; 4 Division of Molecular Immunology, Department of Medicine 3-Rheumatology and Immunology, Universitätsklinikum Erlangen, Friedrich-Alexander-Universität Erlangen-Nürnberg 9171, , Erlangen, Germany; 5 Genomics and Transcriptomics Laboratory, Biological and Medical Research Centre (BMFZ), Heinrich Heine University 9170, Düsseldorf, Germany; 6 Institute of Medical Microbiology and Hospital Hygiene, Heinrich Heine University 9170, Düsseldorf, Germany; University of California Davis, Davis, California, USA

**Keywords:** lymphotoxin beta receptor, *Toxoplasma gondii*, adaptive immunity, B cells, bone marrow, peritoneal cavity, B-1 cells

## Abstract

Lymphotoxin β receptor (LTβR/TNFRSF3) signaling plays a crucial role in immune defense. Notably, LTβR-deficient (LTβR^-/-^) mice exhibit severe defects in innate and adaptive immunity against various pathogens and succumb to *Toxoplasma gondii* infection. Here, we investigated the bone marrow (BM) and peritoneal cavity (PerC) compartments of LTβR^-/-^ mice during *T. gondii* infection, demonstrating perturbed B-cell and T-cell subpopulations in the absence of LTβR signaling. *T. gondii* infection disrupted BM lymphopoiesis, depleting early and mature B cells in WT mice, whereas mature B cells remained present in LTβR^-/-^ BM. LTβR^-/-^ BM also exhibited reduced MHCII^+^ monocytes and a plasma cell compartment skewed toward IgM^+^ rather than IgA^+^ cells. In addition, BM Tcell subsets were altered, exhibiting decreased double-negative (CD4^-^/CD8^-^) and increased CD4^+^ and CD8^+^ T-cell frequencies. Analysis of the BM transcriptome revealed diminished interferon responses but an upregulated TNFα-NF-κB signaling signature in uninfected and infected LTβR^-/-^ mice, potentially compensating for the absence of LTβR signaling. LTβR^-/-^ mice displayed an altered B-1a to B-1b ratio and a predominant presence of neutrophils in the PerC. In summary, we identified novel immunological alterations in the BM and PerC compartments of LTβR^-/-^ mice, which suggest new roles for LTβR signaling in B- and T-cell homeostasis, migration, and pathogen defense.

## INTRODUCTION

The LTβR (TNFRSF3) is a member of the tumor necrosis factor receptor superfamily (TNFRSF) and is expressed by a wide variety of cell types including epithelial, endothelial, stromal, and myeloid cells (e.g., monocytes, macrophages [1, 2], neutrophils [1, 3], dendritic cells [DCs; 4–6], and mast cells), but is absent from lymphoid cells (7, 8). Conversely, the two known LTβR ligands, LTα_1_β_2_ and LIGHT (**l**ymphotoxin-like, exhibits **i**nducible expression, and competes with HSV **g**lycoprotein D for **h**erpesvirus entry mediator, a receptor expressed by **T** lymphocytes), are expressed on lymphoid cells, suggesting that LTβR signaling mainly occurs in a paracrine or juxtacrine manner (7, 8). While the lymphotoxin heterotrimer LTα_1_β_2_ is LTβR-specific, the second ligand LIGHT can also bind two other TNFRSF members: herpes virus entry mediator (HVEM) and soluble decoy receptor 3 (DcR3) (7–9). LTβR-induced downstream signaling occurs primarily via the canonical and the alternative nuclear factor “kappa-light-chain-enhancer” of activated B-cell (NF-κB) pathways (8, 10).

The LTβR is essential for the development of lymphoid organs during embryogenesis and for structural maintenance of lymphoid organs as well as immune cell homeostasis in adulthood (11–13). LTβR-deficient mice (LTβR^-/-^) lack lymph nodes (LN) and Peyer’s Patches (PPs) (11), and exhibit an altered splenic microenvironment characterized by disrupted B- and T-cell compartmentalization (14, 15), germinal center (GC) formation (16, 17), and markedly reduced numbers of follicular DCs (FDCs) (5, 13, 18, 19), macrophages (11, 13, 20, 21), natural killer (NK), and NKT cells (13, 22–25). Another affected organ is the thymus, where LTβR signaling is involved in thymocyte migration and the establishment of central tolerance (26). In addition, LTβR^-/-^ mice exhibit signs of autoimmunity, including splenomegaly, increased perivascular lymphocytic infiltrations of non-lymphoid organs, autoantibody production, and increased baseline immune activation (11, 13, 25, 27–29). Furthermore, the well-documented inability of LTβR^-/-^ mice to mount adequate immune responses against various pathogens underscores its critical role for immunity (13, 25, 29–34). Consequently, there has been growing interest in exploring the role of LTβR signaling in infection, autoimmune diseases, and cancer (35–38).

*Toxoplasma gondii* is an obligate intracellular protozoan parasite that belongs to the phylum Apicomplexa. *T. gondii* can infect virtually all warm-blooded vertebrates, including humans. This successful parasite has infected 30%–50% of the world’s human population (39) and can persist lifelong in the host (40, 41). While *T. gondii* infection usually causes mild, flu-like symptoms in immunocompetent hosts, an infection of immunocompromised individuals or congenitally infected unborn children can lead to diverse and severe health issues, including encephalitis, myocarditis, pneumonia, and abortion (42). No vaccine is currently available against *T. gondii* (43).

The host immune response against *T. gondii* requires a coordinated interplay of innate, adaptive, and cell-autonomous immunity (44–47). *T. gondii* infection stimulates a proinflammatory cascade resulting in massive interferon-gamma (IFNγ) production by NK cells (48, 49), CD4^+^ and CD8^+^ T cells (48, 50–52), and innate lymphoid type I cells (ILC1s) (53–56). An early and potent production of IFNγ is essential for effective parasite control (57, 58). IFNγ (further) activates immune cells such as monocytes, macrophages, DCs, NK, and T cells, and it induces the expression of hundreds of genes involved in cell-autonomous immunity to inhibit intracellular pathogens (47). This includes the direct targeting and destruction of *T. gondii* and its surrounding parasitophorous vacuole (PV) by immunity-related GTPases (IRGs) (59–63) and guanylate-binding proteins (GBPs) (64–68). However, while a strong T-helper 1 (Th1) response driven by IL-12 and IFNγ is necessary for effective parasite restriction, it can also trigger severe immunopathology in the host (69–74).

In addition to classic CD4^+^ and CD8^+^ T-cell populations, double-negative (DN; CD4^-^/CD8^-^) T cells lack both CD4 and CD8 co-receptors and can be found as a rare T-cell subpopulation in the periphery (1%–5% of CD3^+^ T cells) (75, 76). They are enriched in the bone marrow (BM), where they constitute up to one-third of CD3^+^ T cells (77). While details remain elusive, the heterogeneous DN T cells can be of thymic or extrathymic origin and exert proinflammatory as well as immunosuppressive effects (78). Their potential role in immune responses, disease, and therapy has increasingly gained recognition (78–81).

B cells and antibody-mediated immunity further contribute to host defense against *T. gondii* infection (82–89). However, there are major differences between B-cell subpopulations. B-1 cells are mainly of fetal origin and have self-renewing capacities, whereas conventional (B-2) cells need to be continuously generated from progenitors in the adult BM (90). Most B-2 cells leave the BM at the transitional stage and complete their maturation in secondary lymphatic organs like the spleen, from where they may circulate through the bloodstream and re-enter the BM as recirculating mature B cells (91, 92). While B-1 cells are a rare subpopulation in the BM, peripheral blood (PB), spleen, and lymph nodes (0.1%–2% of CD19^+^ B cells), they are enriched in body cavities such as the peritoneal and pleural cavities (35%–70% of CD19^+^ B cells) (93). They are considered innate-like B cells due to their more restricted B-cell receptor repertoire, their role as the major source of natural antibodies, and their capability of rapid, mainly T-cell-independent secretion of low-affinity IgM during early infection (94–99). Depending on the cell surface expression of CD5, B-1 cells are further divided into B-1a (CD5^+^) cells, which are considered the main natural antibody producers, and B-1b (CD5^-^) cells, which are more involved in the adaptive antibody response than B-1a cells (96). While B-1 cells have been shown to contribute to protection against *T. gondii* alongside conventional B-2 cells (86, 89), they are also a predisposed source of autoantibodies induced by *T. gondii* infection and can negatively impact host defense (100, 101). However, the interplay between protozoan parasites and the B-cell response is diverse and not fully understood (102–105).

In our previous studies, we showed that LTβR^-/-^ mice are highly susceptible and rapidly succumb to *T. gondii* (25, 29). The expression of IFNγ and downstream effector molecules, such as mGBPs, was significantly delayed. Adaptive immunity of LTβR^-/-^ mice was also affected, as evidenced by reduced secretion of IFNγ by CD4^+^ T cells and cytotoxic granules by CD8^+^ T cells after *ex vivo* stimulation (25). B cells in the spleen of WT mice were significantly reduced during *T. gondii* infection, but this reduction was attenuated in LTβR^-/-^ mice. In addition, LTβR^-/-^ mice showed only low amounts of parasite-specific IgM in the serum, whereas parasite-specific immunoglobulin heavy-chain class-switching and IgG production were abrogated (25).

Here, we expand and deepen our investigation of the B-cell immune response and B-cell subpopulations to the BM and peritoneal cavity (PerC) of LTβR^-/-^ mice and demonstrate its intriguing dynamics during *T. gondii* infection. A striking phenotype of the LTβR^-/-^ BM was increased mature B-cell frequencies, which displayed a unique resistance to inflammation-induced reduction compared to WT mature B cells. While MHCII^+^ proinflammatory monocytes were diminished in LTβR^-/-^ compared to WT BM, plasma cell (PC) frequencies were comparable or even increased; however, the majority of LTβR^-/-^ bone marrow PCs (BMPCs) displayed surface IgM rather than IgA, in contrast to WT BMPCs. In addition, we found that T-cell subset frequencies were altered in the BM of LTβR^-/-^ mice, with decreased DN T cell and increased CD4^+^ and CD8^+^ T cell frequencies. RNA sequencing revealed significantly reduced interferon-related gene sets and an enriched “TNFα-signaling via NF-κB” gene set in LTβR^-/-^ BM compared to WT BM prior to and after *T. gondii* infection. In the LTβR^-/-^ PerC, B-2 and B-1b cell frequencies were increased, whereas B-1a frequencies were comparable to WT counterparts, leading to altered B-1a to B-1b ratios in LTβR^-/-^ mice. Finally, the predominant presence of neutrophils over T cells in the LTβR^-/-^ PerC on day 9 p.i. marks a profound difference, showing that parasite replication is not effectively inhibited despite neutrophil accumulation, which likely contributed to exacerbated immunopathology. These results will hopefully enhance our understanding of the role of LTβR signaling in immune cell homeostasis and during intracellular parasite infections.

## RESULTS

### Mature B cells are increased in the BM of LTβR^-/-^ mice and show less severe reduction during *T. gondii* infection

**Fig 1:**
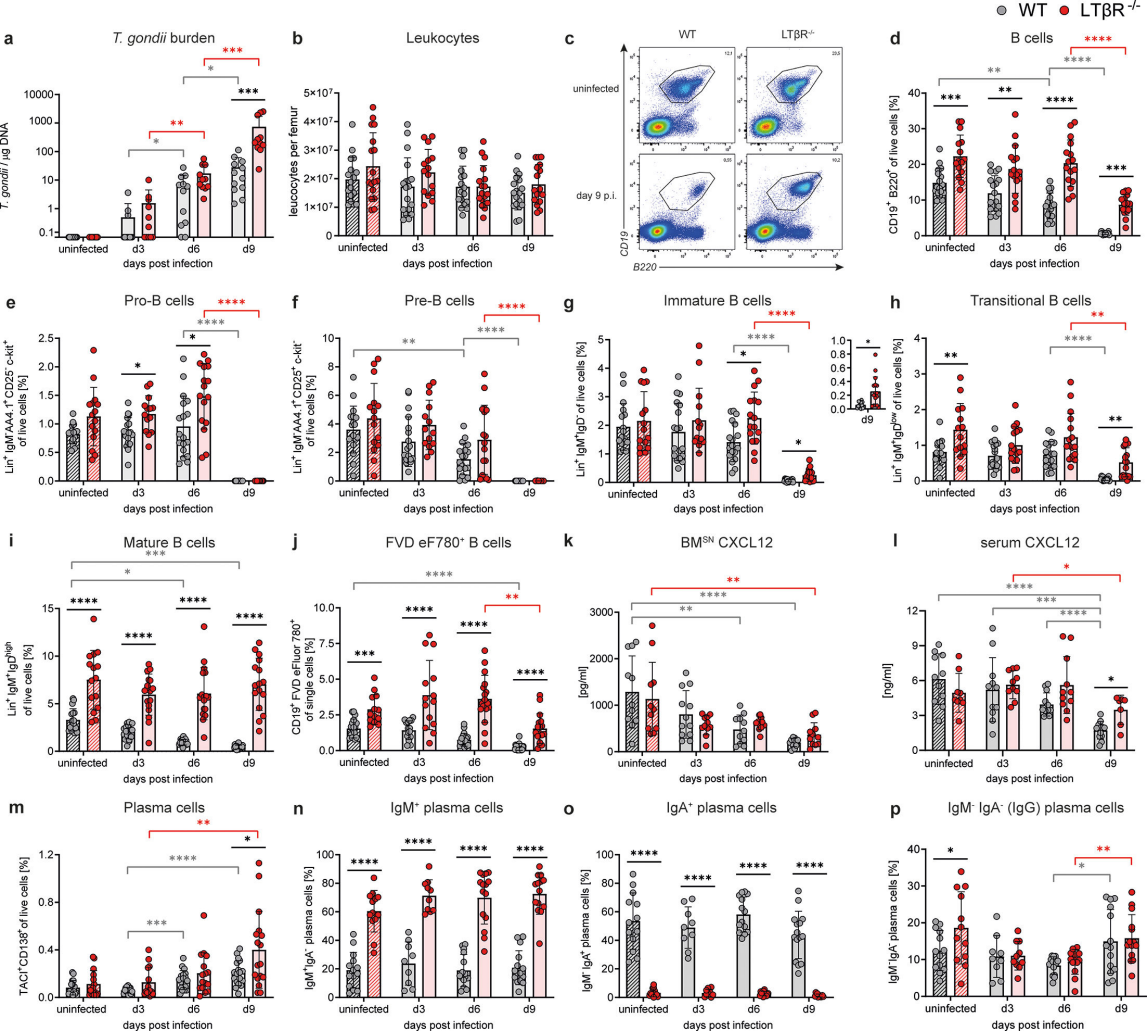
Altered B-cell subpopulations and CXCL12 concentrations in the BM of *T. gondii*-infected WT and LTβR^-/-^ mice. (**a**) DNA was isolated from the BM and used to assess the *T. gondii* burden via quantitative real-time PCR of the B-1 gene (*TgB1*). A standard curve generated from a defined number of ME49 tachyzoites (2914 ± 214 /µL) was used to calculate parasite loads in WT (*n* ≥ 10/group) and LTβR^-/-^ (*n* ≥ 8/group) mice. (**b**) Counted leukocytes per femur from uninfected and infected WT (*n* ≥ 17/group) and LTβR^-/-^ (*n* ≥ 15/group) mice. Using surface marker staining and flow cytometry (gating strategy: Fig. S1), the following immune cell populations in the BM of WT (*n* ≥ 15/group) and LTβR^-/-^ (*n* ≥ 13/group) mice were identified and quantified as percentages of live cells, unless otherwise specified: (**c and d**) Pan-B cells (CD19^+^ B220^+^) (**c**) shows a set of representative images. (**c–i**) Lin^+^ = CD19^+^ B220^+^. (**e**) Pro-B cells (Lin^+^ IgM^-^ AA4.1^+^ CD25^−^ c-kit^+^), (**f**) Pre-B cells (Lin^+^ IgM^-^ AA4.1^+^ CD25^+^ c-kit^-^), (**g**) Immature B cells (Lin^+^ IgM^+^ IgD^−^), (**h**) Transitional B cells (Lin^+^ IgM^+^ IgD^low^), and (**i**) Mature B cells (Lin^+^ IgM^+^ IgD^high^). (**j**) Dead B cells (CD19^+^ FVD eFluor780^+^), % of single cells. (**k and l**) CXCL12 measured in the (**k**) BM^SN^ and (**l**) serum of uninfected and infected WT (*n* ≥ 10/group) and LTβR^-/-^ (*n* ≥ 7/group) mice via bead-based immunoassay (LegendPlex, BioLegend, USA). (**m**) BMPCs (TACI^+^ CD138^+^). WT: *n* ≥ 13/group, LTβR^-/-^: *n* ≥ 14/group. (**n–p**) IgM^+^, IgA^+^, and IgM^-^ IgA^-^ (IgG) expressing PCs (surface Ig), % of BMPCs. WT: *n* ≥ 9/group, LTβR^-/-^: *n* ≥ 10/group. All data shown represent at least four independent experiments; symbols represent individual animals and columns represent means ± SD. *P  <  0.05; **P  <  0.01; ***P  <  0.001; ****P  <  0.0001.

WT and LTβR^-/-^ mice were intraperitoneally (i.p.) infected with *T. gondii* (ME49) and analyzed on days 3, 6, and 9 p.i. In the BM, *T. gondii* burden increased over the course of infection; on day 9 p.i., LTβR^-/-^ mice showed significantly increased *T. gondii* numbers (>20-fold) as compared to WT mice (Fig. 1a). At the same time, total leukocyte numbers per femur did not significantly differ or change between genotypes over the course of *T. gondii* infection (Fig. 1b). Interestingly, CD19^+^ B220^+^ pan-B cells were already significantly increased in LTβR^-/-^ BM before infection compared to WT BM (Fig. 1c and d; for gating strategy see Fig. S1, for absolute cell numbers see S2a). But while pan-B-cell frequencies were found significantly reduced in WT BM by day 6 p.i., they remained stable in LTβR^-/-^ BM at this point (Fig. 1c and d). Both genotypes showed a significant reduction of pan-B cells from day 6 to day 9 p.i. However, the overall pan-B-cell reduction was much less severe in LTβR^-/-^ mice (−61.0% on day 9 p.i. compared to uninfected) compared to WT mice (−95.7% on day 9 p.i. compared to uninfected), mirroring the course of splenic B-cell frequencies after *T. gondii* infection as described previously (25).

To identify which B-cell subpopulation(s) was responsible for the increased B-cell numbers in the LTβR^-/-^ BM, B-cell developmental stages were investigated based on surface marker expression (Fig. S1; Table S1 and S2). Pro-, pre-, and immature B-cell populations were mostly comparable between genotypes (Fig. 1e through g; Fig. S2b through d), while transitional B-cell (Lin^+^ IgM^+^ IgD^low^, Fig. 1H; Fig. S2e) frequencies were increased in uninfected LTβR^-/-^ mice and on day 9 p.i. Notably, mature B cells (Lin^+^ IgM^+^ IgD^high^) were the primary contributors to the increased BM B-cell population in LTβR^-/-^ mice, as they were significantly increased at all examined time points compared to their WT BM counterparts (Fig. 1i; Fig. S2f).

The strong reduction of B cells on day 9 p.i. was observed across all investigated B-cell subpopulations in WT BM (Fig. 1e through i; Fig. S2b through f). But while this was also evident in early-stage B-cell populations in LTβR^-/-^ BM, the transitional and mature B cells were notable exceptions. More precisely, on day 9 p.i., pro-, pre-, and immature B cells were nearly absent in both WT and LTβR^-/-^ BM (Fig. 1e through g; Fig. S2b through d). However, while transitional B cells in LTβR^-/-^ mice were significantly reduced on day 9 compared to day 6 p.i., they were significantly increased compared to WT on day 9 p.i. (Fig. 1h; Fig. S2e). Mature B cells remained unaffected in LTβR^-/-^ BM during infection and were therefore highly significantly increased compared to the WT at all time points during infection (Fig. 1i; Fig. S2f). These results demonstrate that both phenotypes observed in the LTβR^-/-^ BM - the increased B cells under steady-state conditions and the enhanced resistance to reduction during *T. gondii* infection - are specific to particular late-stage B-cell populations, with mature B cells exhibiting the most significant alterations.

To investigate whether the reduction of B cells on day 9 p.i. was caused by B-cell death or by mobilization and egress, we used a viability dye (FVD eF780) to detect dead cells (Fig. S1; Table S1 and S2). LTβR^-/-^ mice had significantly increased frequencies of dead B cells in the BM before and during *T. gondii* infection (Fig. 1j), even though both genotypes showed comparable frequencies of dead BM leukocytes (Fig. S2g). On day 9 p.i., frequencies of dead B cells were lower compared to previous days, possibly due to the overall reduced presence of B cells on that day in both genotypes (Fig. 1j). Similar to the results described for living cells, dead early B cells (FVD eF780^+^ CD19^+^ AA4.1^+^ IgM^-^) were almost completely absent on day 9 p.i. in both WT and LTβR^-/-^ animals (Fig. S2h), while dead mature B cells (FVD eF780^+^ CD19^+^ IgM^+^ IgD^+^) were significantly increased in LTβR^-/-^ animals (Fig. S2i).

The chemokine C-X-C motif chemokine 12 (CXCL12) is, among other functions, essential for hematopoiesis and regulating B-cell development by directing them to and retaining them in specific BM niches (106). During inflammation, a reduction of CXCL12 in the BM has been shown to lead to the mobilization and egress of B-cell precursors (107). CXCL12 concentrations were therefore determined in the BM supernatant (BM^SN^) and serum of WT and LTβR^-/-^ mice. CXCL12 concentrations declined during *T. gondii* infection and were significantly reduced in the BM^SN^ (Fig. 1k) and serum (Fig. 1l) on day 9 p.i. in WT and LTβR^-/-^ mice. While the decrease in BM^SN^ CXCL12 after infection likely explains the egress of early B-cell populations and thus the almost complete loss of pro- and pre-B cells observed on day 9 p.i., early B cells (AA4.1^+^) were decreased, rather than increased, in the PB on day 9 p.i. (Fig. S3a). Instead, a significantly increased AA4.1-positive myeloid (CD11b^+^) population was identified in the PB of both genotypes (Fig. S3b and c). Overall, LTβR^-/-^ mice showed higher numbers of circulating PB leukocytes (Fig. S3d) and increased frequencies of PB (mature) B cells (Fig. S3e and f) compared to WT controls. In contrast to the unaffected frequencies of mature B cells in the LTβR^-/-^ BM, their frequency in the PB was reduced on day 9 p.i. but was still significantly higher than in WT animals (Fig. S3f).

In contrast to CXCL12, concentrations of B-cell activating factor (BAFF), another cytokine important for B-cell development, remained stable in the BM^SN^ (Fig. S4a) and increased in the serum (Fig. S4b) over the course of infection. However, BAFF concentrations were always comparable between WT and LTβR^-/-^ mice.

Surprisingly, despite lacking lymph nodes, PPs (11), and GC reactions (13, 17), LTβR^-/-^ mice had equal or increased (day 9 p.i.) frequencies of BMPCs (TACI^+^ CD138^+^ [108]) compared to WT mice (Fig. 1m, Fig. S4c and d), the origin of which remains unclear. However, the majority of LTβR^-/-^ BMPCs were surface IgM positive, which is in contrast to the predominantly IgA-positive WT BMPCs (Fig. 1n and o; Fig. S4e and g). Double-negative (IgM^−^ IgA^−^), presumably IgG^+^, BMPC frequencies were elevated in uninfected LTβR^-/-^ mice but otherwise comparable between genotypes (Fig. 1p; Fig. S4h).

In summary, the LTβR^-/-^ BM contained significantly increased frequencies of mature B cells under steady-state conditions, which were also more resistant to reduction during *T. gondii* infection compared to WT counterparts. However, frequencies of earlier B-cell developmental stages were comparable between genotypes, which both exhibited a significant reduction in CXCL12 concentrations in the BM^SN^ over the course of infection.

### Characterization of T-cell, dendritic-cell, NK-cell, and proinflammatory monocyte/macrophage populations in the LTβR^-/-^ BM

Next, a detailed analysis of BM immune cell populations, such as T cell, DC, and NK cell (sub)populations was conducted (Table S1 and S2; for gating strategy, see Fig. S5 and S6; absolute cell numbers are given in Fig. S7).

**Fig 2:**
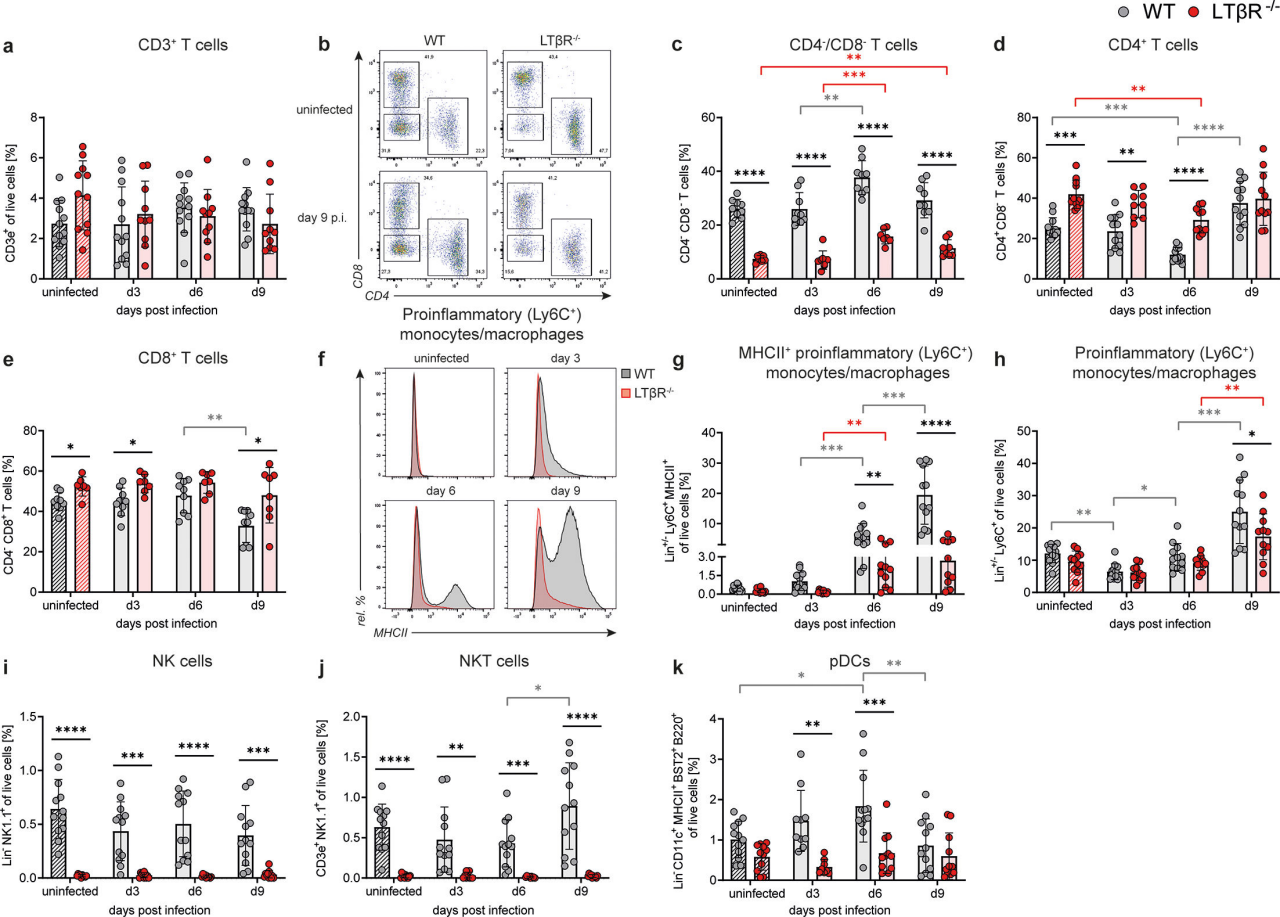
Altered T cells, proinflammatory monocytes/macrophages, NK cells, NKT cells, and pDCs in the BM of *T. gondii*-infected LTβR^-/-^ mice. Using surface marker staining and flow cytometry, the following immune cell populations (gating strategies: Fig. S5 and S6) in the BM of WT and LTβR^-/-^ mice were identified and quantified as percentages of live cells, unless otherwise specified: (**a**) T cells (CD3e^+^) (**b–e**) (**b**) shows a set of representative images of the gating of (**c**) double-negative (CD4^-^ CD8^-^), (**d**) CD4^+^, and (**e**) CD8^+^ T cells, all shown as % of CD3e^+^ T cells. WT: *n* ≥ 9/group and LTβR^-/-^: *n* ≥ 7/group. (**f**) Representative histograms of MHCII expression (%) on proinflammatory monocytes/macrophages. Each curve is scaled to 100%. (**g**) MHCII-positive proinflammatory monocytes/macrophages (CD19^-^ CD3e^-^ NK1.1^-^ CD11b^+^ Ly6G^-^ Ly6C^+^ MHCII^+^). (**h**) Proinflammatory monocytes/macrophages (CD19^-^ CD3e^-^ NK1.1^-^ CD11b^+^ Ly6G^-^ Ly6C^+^). (**i**) NK cells (CD19^-^ CD3e^-^ NK1.1^+^). (**j**) NKT cells (CD3e^+^ NK1.1^+^). (**k**) Plasmacytoid DCs (pDCs; CD19^-^ CD3e^-^ NK1.1^-^ CD11c^+^ CD11b^-^ MHCII^+^ B220^+^ BST-2^+^). All data shown represent at least four independent experiments; symbols represent individual animals and columns represent mean values ± SD. *P  <  0.05; **P  <  0.01; ***P  <  0.001; ****P  <  0.0001.

BM T-cell frequencies (CD3e^+^) were comparable between both genotypes and were not significantly altered during *T. gondii* infection (Fig. 2A Fig. S7a). Interestingly, T-cell subset frequencies differed significantly between both genotypes: double-negative (DN; CD4^-^/CD8^-^) T cells were twofold to threefold lower, while CD4^+^ and CD8^+^ T-cell frequencies were higher in LTβR^/^ BM compared to WT BM, both prior to and during *T. gondii* infection (Fig. 2b through e). These genotype-dependent differences were also partially observed in the absolute cell numbers for DN T cells and CD4^+^ T cells but were not statistically significant for CD8^+^ T cells (Fig. S7b through d).

In addition to B- and T-cells, a striking phenotype was the reduced upregulation of surface major histocompatibility complex class II (MHCII) on proinflammatory (Ly6C^+^) monocytes/macrophages (defined as CD3e^-^ CD19^-^ NK1.1^-^ CD11b^+^ Ly6G^-^ Ly6C^+^) in the BM of LTβR^-/-^ mice (Fig. 2f and g; Fig. S7e). A similar reduction was found in the PB (Fig. S7f) but not in the PerC (Fig. S7g; for gating strategy, see Fig. S8), where MHCII-expressing proinflammatory monocyte/macrophage frequencies were comparable between genotypes. Notably, the overall frequencies of proinflammatory monocytes/macrophages were similar between LTβR^-/-^ and WT BM, except on day 9 p.i., where a significant reduction was observed in LTβR^-/-^ BM (Fig. 2h). However, this difference was not reflected in the absolute cell numbers (Fig. S7h).

LTβR signaling is also essential for the generation of functional NK cells (13, 109), NKT cells (13, 22, 23), as well as DCs (5, 13, 110). We can confirm these findings in the BM compartment of LTβR^-/-^ mice, where basically neither NK (Fig. 2i; Fig. S7i) nor NKT cells (Fig. 2j; Fig. S7j) were detectable before and during *T. gondii* infection, and where pDCs were reduced particularly on day 6 p.i. compared to WT mice (Fig. 2k; Fig. S7k). Notably, BM neutrophil frequencies (Fig. S7l) and numbers (Fig. S7m) did not significantly differ between genotypes. These results show that the absence of LTβR signaling perturbed specific myeloid and lymphoid immune cell populations in the BM.

### BM RNA sequencing reveals impaired upregulation of IFNγ- and IFNα-related gene sets concurrent with enriched TNFα signaling and an increased presence of eosinophil-associated transcripts in LTβR^-/-^ mice

**Fig 3:**
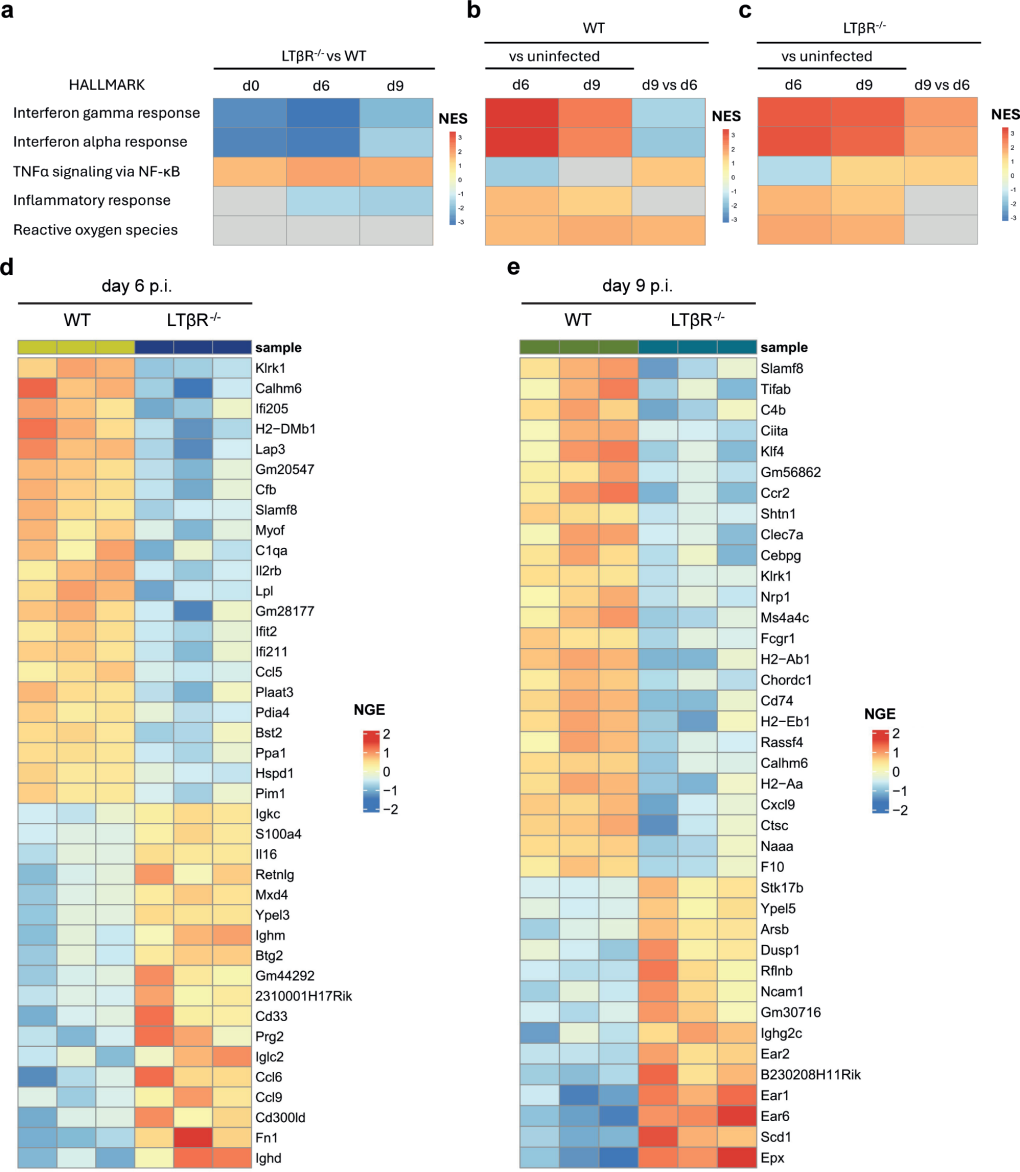
LTβR^-/-^ BM shows less enrichment of interferon-related gene sets compared to WT BM, but more eosinophil-related gene expression on day 9 p.i. (**a–c**) Summarized results of the gene set enrichment analysis (GSEA) of mRNA sequencing data from BM samples of uninfected and *T. gondii*-infected WT (*n* = 3/group) and LTβR^-/-^ mice (*n* = 3, except for LTβR^-/-^ uninfected: *n* = 2). Normalized enrichment scores (NES) of selected GSEA hallmark gene sets (see Fig. S9a through d and S10 for complete GSEA data) are represented as colors in a heat map. A positive NES value indicates gene set enrichment in the experimental condition, a negative NES value indicates gene set enrichment in the control condition, and a gray-colored tile describes non-significance (adjusted *P*-value > 0.01). The (**a**) left panel summarizes genotype comparisons (LTβR^-/-^ vs. WT), displaying enrichment in LTβR^-/-^ compared to WT controls. The (**b**) middle panel summarizes intra-WT comparisons: d6 and 9 p.i. vs. uninfected controls, displaying enrichment in infected animals; and d9 vs. d6, displaying enrichment on day 9 p.i. compared to day 6 p.i. Similarly, the (**c**) right panel summarizes intra-LTβR^-/-^ comparisons: d6 and 9 p.i. vs. uninfected controls, displaying enrichment in infected animals; and d9 vs. d6, displaying enrichment on day 9 p.i. compared to day 6 p.i. (**d and e**) Heat map of the top 40 differentially expressed genes (based on the adjusted *P*-values) in WT and LTβR^-/-^ BM on (**d**) day 6 p.i and (**e**) day 9 p.i., sorted by upregulation and downregulation. NGE = normalized gene expression.

Sequencing of mRNA (RNASeq) of WT and LTβR^-/-^ BM was performed to investigate the BM transcriptome during *T. gondii* infection. Gene set enrichment analysis (GSEA) was employed to compare gene sets between genotypes (LTβR^-/-^ vs WT, Fig. 3a; Fig. S9) and across the infection timeline (d6 and d9 p.i. vs. uninfected controls, and d9 vs. d6 p.i., Fig. 3b and c; Fig. S10).

In the genotype comparison (Fig. 3a), the gene sets “Interferon gamma response” and “Interferon alpha response” were significantly enriched in the BM of all WT cohorts compared to LTβR^-/-^ counterparts, particularly in uninfected controls and on day 6 p.i. By contrast, the “TNFα signaling via NF-κB” gene set was significantly enriched in LTβR^-/-^ BM at all time points examined. Other inflammation-related gene sets, such as “Inflammatory response” and “Reactive oxygen species,” were enriched in WT BM or showed no significant difference between genotypes.

When comparing each infected genotype to its respective uninfected control, both WT (Fig. 3b) and LTβR^-/-^ (Fig. 3c) BM exhibited proinflammatory gene set enrichment, particularly “Interferon gamma response” and “Interferon alpha response,” on days 6 and 9 p.i. Notably, these gene sets were less enriched in WT BM on day 9 p.i. compared to day 6 p.i. (Fig. 3b), whereas LTβR^-/-^ BM showed continued enrichment from day 6 to day 9 p.i. (Fig. 3c). The “TNFα signaling via NF-κB,” “Inflammatory response,” and “Reactive oxygen species” gene sets were enriched in the BM of both genotypes during *T. gondii* infection (Fig. 3b and c). A summary of additional GSEA gene sets is provided in Fig. S9d.

Thus, compared to WT BM, the LTβR^-/-^ BM exhibited reduced enrichment of the critical “Interferon gamma response” and “Interferon alpha response” gene sets but showed greater enrichment of the “TNFα signaling via NF-κB” pathway. Overall, both genotypes displayed increased expression of IFN-related gene sets over the course of *T. gondii* infection, with expression peaking in WT BM on day 6 p.i. but continuing to increase until day 9 p.i. in LTβR^-/-^ BM.

We also identified the top differential transcripts between WT and LTβR^-/-^ BM on days 6 and 9 p.i. Notably, LTβR^-/-^ BM displayed elevated expression of immunoglobulin genes (*Igkc*, *Ighm*, *Iglc2,* and *Ighd*) and specific migration/adhesion genes (*Ccl6*, *Ccl9,* and *Fn1*), but lower expression of IFNγ-inducible genes (*Ifi205*, *Ifit2*, *Ifi211,* and *Bst2*) compared to WT BM on day 6 p.i. (Fig. 3d). On day 9 p.i., transcripts of several members of the eosinophil-associated ribonuclease A family (*Ear1*, *Ear2,* and *Ear6*) and *Epx* (eosinophil peroxidase) were significantly increased in LTβR^-/-^ BM (Fig. 3e), whereas expression of genes associated with MHCII and antigen presentation (*Ciita*, *H2-Ab1*, *H2-Aa*, *H2-Eb1,* and *CD74*), immune cell function (*Klrk1* and *Fcgr1*), and migration (*Ccr2* and *Cxcl9*) was decreased compared to WT BM (Fig. 3e). *Slamf8* transcripts, which are expressed by a variety of activated myeloid cells, including IFNγ-activated monocytes and macrophages (111), were reduced on both days in LTβR^-/-^ BM. Interestingly, only a small number of *T. gondii*-derived transcripts were detected, with the majority being identified in LTβR^-/-^ BM on day 9 p.i., primarily encoding GRA (*T. gondii* dense granule organelle) proteins (Fig. S9e).

### Leukocyte counts**,** *T. gondii* burden, and dead cell frequencies are elevated in the PerC of LTβR^-/-^ mice

**Fig 4:**
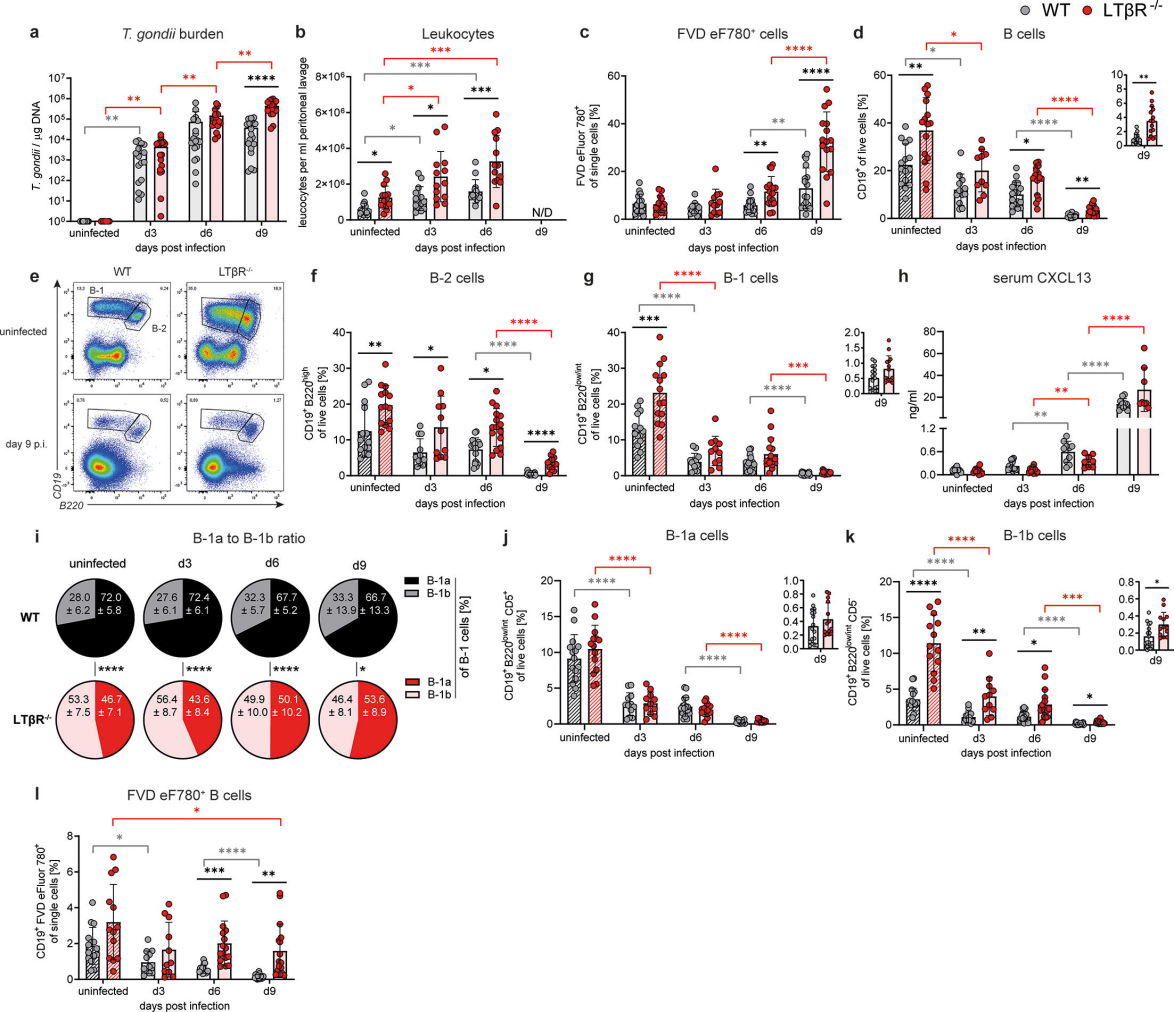
Altered B-cell subpopulations in the PerC of *T. gondii*-infected WT and LTβR^-/-^ mice. (**a**) DNA was isolated from cells obtained by peritoneal lavage of uninfected and *T. gondii-*infected WT (*n* ≥ 14/group) and LTβR^-/-^ (*n* ≥ 12/group) mice and used to assess parasite burden via quantitative real-time PCR of the *T. gondii* B-1 gene (*TgB1*). A standard curve generated from a defined number of ME49 tachyzoites (2,914 ± 214 /µL) was used to calculate parasite loads. (**b**) Counted peritoneal leukocytes per mL lavage from uninfected and infected WT and LTβR^-/-^ mice (both *n* ≥ 13/group). Peritoneal lavage was performed by injection of 5 mL ice-cold PBS, followed by a gentle massage and subsequent recovery of fluid. No reliable cell count can be reported for day 9 p.i. due to large amounts of cell debris and clumped cells. Exclusion of all non-single-cell events during flow cytometry enabled the analysis of the remaining single cells. Using surface marker staining and flow cytometry, the following immune cell populations (gating strategy: Fig. S8) in the PerC of WT (*n* ≥ 12/group) and LTβR^-/-^ (*n* ≥ 11/group) mice were identified and quantified as percentages of live cells, unless otherwise specified: (**c**) Dead leukocytes (FVD eFluor780^+^), % of single cells. (**d**) B cells (CD19^+^). (**e–g**) B-1 (CD19^+^ B220^low/int^) and B-2 (CD19^+^ B220^high^) cells. (**e**) A set of representative images. Please note that the large CD19^-^ B220^+^ cell population in uninfected mice was found to be large CD11b^+^ Ly6G^-^ Ly6C^-^ cells, which represent autofluorescent large tissue-resident macrophages that disappear after infection (112, 113). (**h**) CXCL13 measured in the serum of uninfected and infected WT (*n* ≥ 10) and LTβR^-/-^ (*n* ≥ 8) *via* bead-based immunoassay (LegendPlex, BioLegend, USA). (**i**) Ratios of B-1a (CD19^+^ B220^low/int^ CD5^+^) to B-1b (CD19^+^ B220^low/int^ CD5^-^) cells, % of B-1 cells. (**j and k**) Frequencies of B-1a (CD19^+^ B220^low/int^ CD5^+^) and B-1b (CD19^+^ B220^low/int^ CD5^-^) cells. (**l**) Dead B cells (CD19^+^ FVD eFluor780^+^), % of single cells. All data shown represent at least three independent experiments; symbols represent individual animals and columns represent mean values ± SD. *P  <  0.05; **P  <  0.01; ***P  <  0.001; ****P  <  0.0001.

The number of *T. gondii* tachyzoites in the PerC as the primary site of infection was significantly increased and 10-fold higher in LTβR^-/-^ compared to WT mice on day 9 p.i. (Fig. 4a). Both genotypes exhibited elevated peritoneal leukocyte counts compared to their respective uninfected controls. However, peritoneal leukocyte numbers were significantly higher in LTβR^-/-^ mice compared to WT mice, both before infection and on days 3 and 9 p.i. (Fig. 4b). In addition, most but not all mice of both genotypes developed ascites by day 9 p.i. (data not shown). The frequency of dead cells within the total leukocyte population was found to be comparable between genotypes in uninfected controls and early during infection, but significantly higher in LTβR^-/-^ mice on days 6 and 9 p.i. (Fig. 4c). Due to the higher leukocyte counts in the LTβR^-/-^ PerC, the absolute number of dead cells was increased at all analyzed time points (Fig. S11a). In summary, LTβR^-/-^ mice exhibited increased leukocyte counts coupled with higher parasite burden and elevated dead cells.

### Frequencies of B-2 and B-1b, but not B-1a cells, were increased in the PerC of LTβR^-/-^ mice

Similar to the BM phenotype, B-cell frequencies as well as absolute numbers in the PerC were significantly increased in LTβR^-/-^ mice before and during *T. gondii* infection (Fig. 4d; Fig. S11b). The frequency of peritoneal B cells was significantly reduced on day 9 p.i. in WT (−95.8% on day 9 p.i. compared to uninfected) and, to a slightly lesser extent, in LTβR^-/-^ mice (−90.6% on day 9 p.i. compared to uninfected).

In addition to conventional B-2 cells (CD19^+^ B220^high^), the PerC was enriched with B-1 cells (CD19^+^ B220^low^), a special B-cell subset with distinct origins, phenotypes, and functions (114, 115). Both B-1 and B-2 frequencies were increased in uninfected LTβR^-/-^ mice (Fig. 4e through g, see Fig. S11c and d for absolute cell numbers). During infection, B-2 cell populations initially remained stable but then dropped between days 6 and 9 p.i. (Fig. 4f). By contrast, B-1 cell frequencies already dropped significantly by day 3 p.i. and then again between days 6 and 9 p.i. (Fig. 4g). Serum concentrations of CXCL13, a chemokine essential for B-1 cell functionality and mobility (116, 117), increased during the course of infection and were markedly elevated on day 6 and especially day 9 p.i., but remained comparable between WT and LTβR^-/-^ mice (Fig. 4h). Similar kinetics were observed in the BM^SN^ (Fig. S11e).

B-1 cells can be further classified into B-1a and B-1b cells based on their CD5 surface marker expression (Fig. S11f). The PerC B-1a to B-1b ratio of LTβR^-/-^ mice was roughly 1:1 (with slightly higher B-1b frequencies), which shifted in favor of the B-1a population over the course of infection (Fig. 4i). By contrast, WT B-1 cells mainly consisted of B-1a cells (72.0% ± 5.8 %), which slightly decreased over the course of infection. Importantly, the shift in LTβR^-/-^ B-1a to B-1b frequencies was not caused by a reduction in B-1a but rather an increase in B-1b frequencies, as determined by B-1a and B-1b live cell frequencies (Fig. 4j and k). This was also confirmed through a direct B-1a (CD19^+^ CD5^+^) gating without prior B-1 gating (Fig. S11g and h). Thus, B-1a cell frequencies were comparable, and B-1b cell frequencies were increased in LTβR^-/-^ compared to WT mice. Both B-1 subpopulations showed a biphasic decrease (from uninfected to day 3 and from day 6 to 9 p.i.). While B-1a cell frequencies were comparable between genotypes, the absolute numbers of B-1a cells were higher in the LTβR^-/-^ PerC due to the increased total leukocyte count (Fig. 4b; Fig. S11i and j). Dead peritoneal B cells (FVD eF780^+^) were significantly higher in LTβR^-/-^ mice, particularly on days 6 and 9 p.i. (Fig. 4l; Fig. S11k).

In summary, in LTβR^-/-^ mice, the increased B-2 cell phenotype includes both the BM and PerC compartments. In uninfected LTβR^-/-^ mice, B-1b frequencies were increased and shifted the B-1a to B-1b ratio, whereas B-1a frequencies remained comparable to the WT PerC. After *T. gondii* infection, both genotypes displayed a marked reduction in B-1 cell frequencies on day 3 p.i., which was absent for B-2 cells.

### LTβR^-/-^ PerC immune cell composition is dominated by neutrophils instead of T cells in late acute *T. gondii* infection

**Fig 5:**
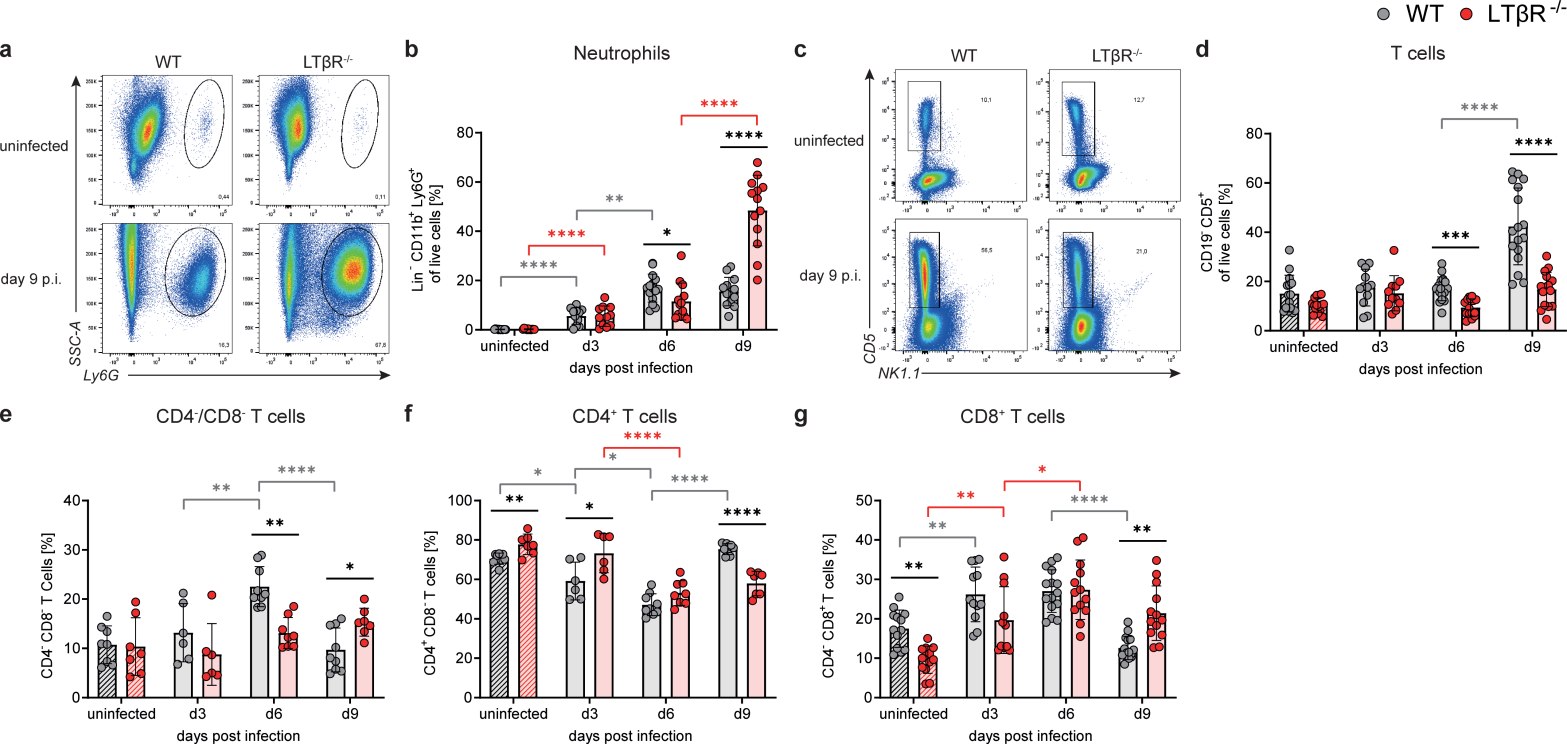
Neutrophils, instead of T cells, dominate the PerC immune cell composition of LTβR^-/-^ mice on day 9 p.i. during *T. gondii* infection**.** Using surface marker staining and flow cytometry, the following immune cell populations (gating strategy: Fig. S8) in the PerC of WT (*n* ≥ 12/group) and LTβR^-/-^ (*n* ≥ 11/group) mice were identified and quantified as percentages of live cells, unless otherwise specified: (**a and b**) neutrophils (CD19^-^ CD5^-^ NK1.1^−^ CD11b^+^ Ly6G^+^). (**a**) A set of representative images. (**c and d**) T cells (CD19^-^ CD5^+^). (**c**) A set of representative images. (**e–g**) Double-negative (CD4^−^ CD8^−^), CD4^+^ and CD8^+^ T cells, % of T cells. WT: *n* ≥ 6/group, LTβR^-/-^: *n* ≥ 6/group. All data shown represent at least three independent experiments; symbols represent individual animals and columns represent mean values ± SD. *P  <  0.05; **P  <  0.01; ***P  <  0.001; ****P  <  0.0001.

Neutrophils were almost absent in the PerC of uninfected controls but increased significantly in both genotypes during *T. gondii* infection (Fig. 5a and b; Fig. S12a). By day 9 p.i., they dominated the peritoneal immune cell composition in LTβR^-/-^ mice (48.4 ± 14.4 %), whereas (CD4^+^) T cells were the major population in WT mice (42.4 ± 15.6 % pan-T cells, Fig. 5c and d). Peritoneal T-cell subset frequencies were more similar between genotypes than those in the BM (Fig. 3a through e) but still showed genotype-specific differences in DN T cells (Fig. 5e), CD4^+^ T cells (Fig. 5f), and CD8^+^ T cells (Fig. 5g). Notably, uninfected LTβR^-/-^ PerCs had increased CD4^+^ and reduced CD8^+^ T-cell frequencies compared to WT mice, indicating altered peritoneal T-cell composition even before infection. By contrast, by day 9 p.i., LTβR^-/-^ PerCs displayed reduced CD4^+^ and higher CD8^+^ T-cell frequencies compared to WT. Regarding absolute cell numbers, DN T cells differed significantly between genotypes in uninfected mice and on day 6 p.i., whereas CD4^+^ T cells and CD8^+^ T-cell numbers showed no statistically significant differences (Fig. S12b through e).

### LTβR^-/-^ mice display increased inflammatory infiltrates in the lung and liver

**Fig 6:**
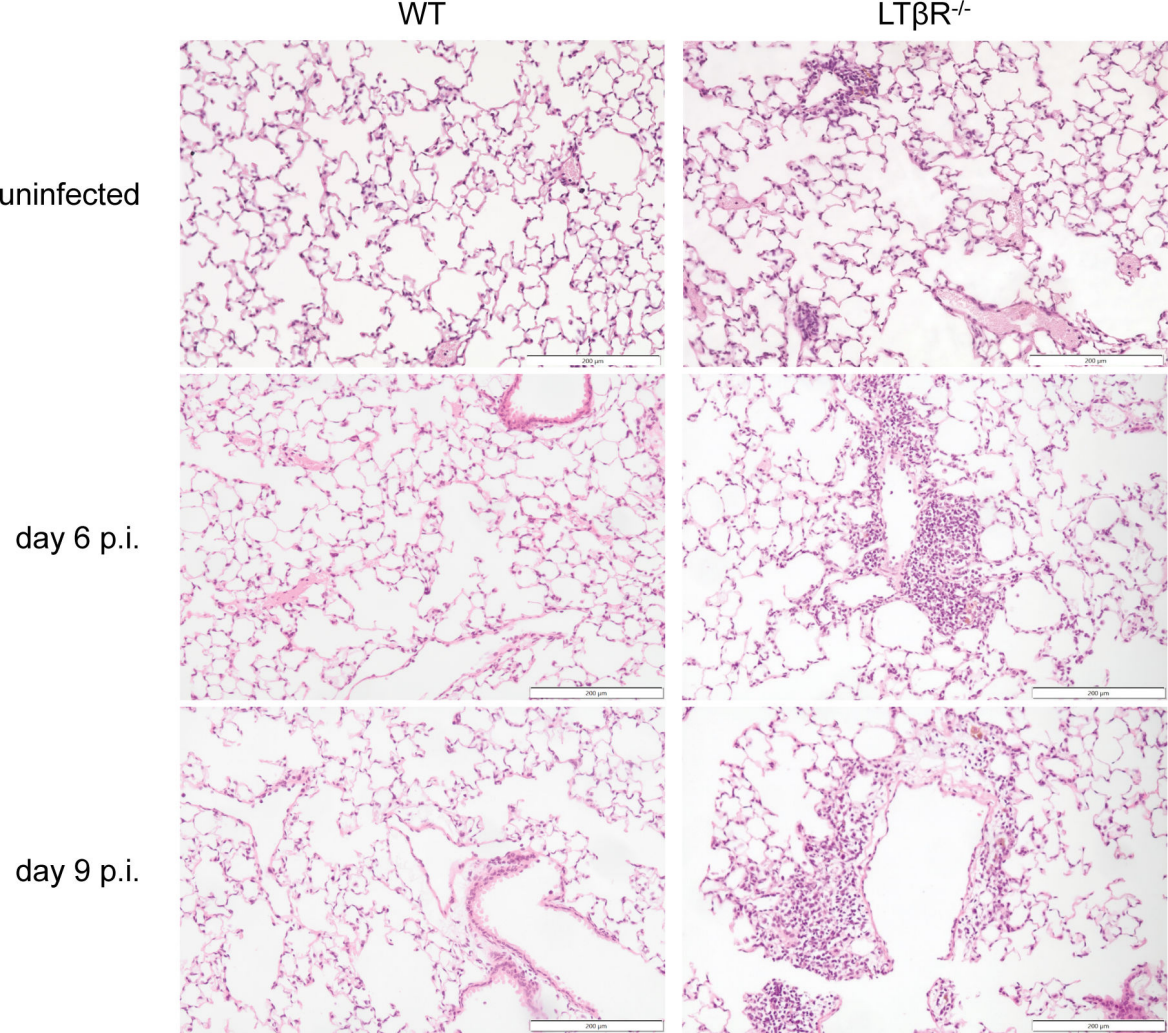
LTβR^-/-^ mice exhibit more inflammatory infiltrations in the lung during *T. gondii* infection. Representative images of H&E-stained lungs from uninfected and *T. gondii*-infected WT and LTβR^-/-^ mice. *n* = 3 except for LTβR^-/-^ uninfected (*n* = 1). Scale bar: 200 µm.

A necropsy of lung, liver, brain, kidney, spleen, and BM, followed by histological analysis, was performed to further investigate the impact of LTβR deficiency during *T. gondii* infection. LTβR^-/-^ mice are known to exhibit abnormal lymphocytic infiltrations in the lung even in the absence of infection (11, 13, 118), a finding also evident in our histological analysis (Fig. 6). *T. gondii* infection increased lung infiltration compared to uninfected controls, with LTβR^-/-^ mice exhibiting more pronounced inflammatory infiltrates than WT mice, consistent with their baseline phenotype. After infection, WT animals showed minimal pulmonary pathology, characterized by mild, scattered monocytic infiltration on day 9 p.i., whereas LTβR^-/-^ lungs displayed increased perivascular and pleural mixed inflammatory infiltrates on days 6 and 9 p.i. (Fig. 6).

**Fig 7:**
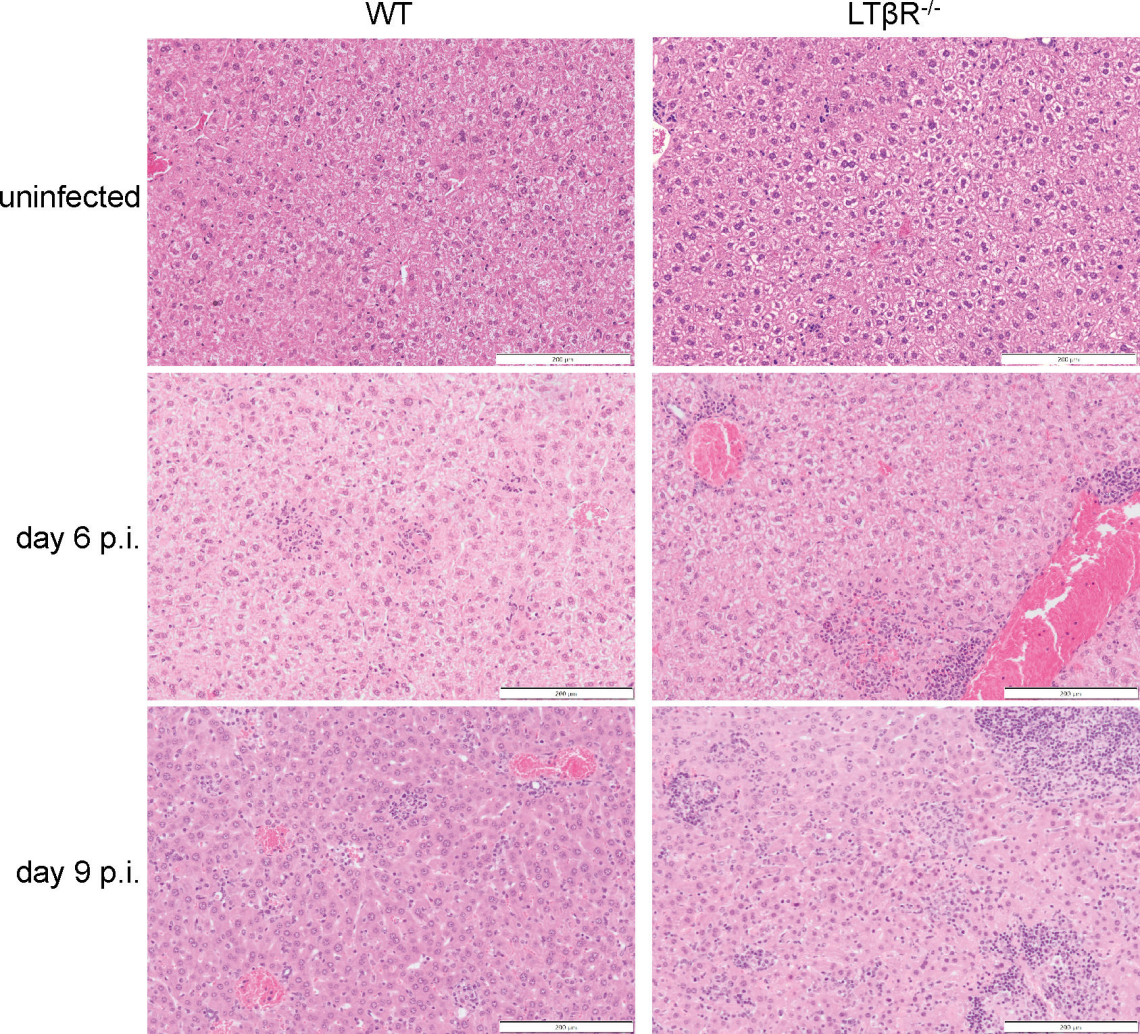
LTβR^-/-^ mice exhibit more inflammatory infiltrations in the liver during *T. gondii* infection. Representative images of H&E-stained livers from uninfected and *T. gondii*-infected WT and LTβR^-/-^ mice. *n* = 3 except for LTβR^-/-^ uninfected (*n* = 1). Scale bar: 200 µm.

*T. gondii* infection also led to more pronounced inflammatory infiltrates in the liver of LTβR^-/-^ compared to WT mice, mirroring the lung phenotype. The liver of WT mice displayed multifocal inflammatory foci occasionally associated with necrotic cells, as well as marked capsular and minimal perivascular/periportal inflammation (Fig. 7). On day 6 p.i., these inflammatory/necrotic foci were few, scattered, and dominated by neutrophils and cellular debris (acute necrosis); on day 9 p.i., foci were more numerous, with often macrophages predominating (subacute necrosis). By contrast, the livers of LTβR^-/-^ mice displayed more extensive inflammation, with predominantly (sub)capsular, perivascular, and mixed mononuclear infiltrates, and randomly scattered areas of multiple hepatocellular necrosis and neutrophilic inflammation. Large numbers of monocytes were usually visible within the lumen of large-caliber vessels (monocytic leukocytosis). On day 6 p.i., inflammatory foci were few and <50% of vessels were affected. On day 9 p.i., most vessels had perivascular mixed mononuclear inflammation, parenchymal foci were increased in number, and macrophages predominated (subacute necrosis).

Neither the brain nor BM displayed genotype-specific differences in the histological analysis (data not shown). WT and LTβR^-/-^ BM had mild to moderately increased granulopoiesis on days 6 and 9 p.i., as evident by increased ratios of myeloid to erythroid cells (data not shown). The kidneys displayed mild to moderate capsular inflammatory infiltrations, but no genotype-specific differences (Fig. S13).

## DISCUSSION

B cells play a crucial role in both protective and, counterintuitively, sometimes in disease-aggravating responses during protozoan parasite infections (83, 87, 100, 101). LTβR^-/-^ mice, which exhibit high susceptibility to *T. gondii* infection, show a compromised ability to generate parasite-specific IgM and IgG responses (25). In this study, we investigated the different immune cell compartments (with a focus on B cells) in homeostasis and the impact of absent LTβR signaling on B-cell populations in the BM and PerC of LTβR^-/-^ mice during *T. gondii* infection.

In adult mice, B-2 cell development initially occurs in the BM, with most immature B cells then migrating to the spleen to complete their development, while some mature within the BM itself (91, 119, 120). Uninfected LTβR^-/-^ mice exhibited an increased number of B cells in the BM compared to WT mice, with mature B cells (IgD^high^) being identified as the predominant subset. In the BM, these recirculating mature B cells normally reside in extravascular perisinusoidal niches alongside T cells, monocytes, and BMDCs, where they receive survival signals such as B cell-activating factor (BAFF) and macrophage migration inhibitory factor (MIF), and contribute to immune responses against blood-borne pathogens (120–122). A disruption of this niche, particularly alterations in the expression of membrane-bound and secreted molecules involved in chemotaxis and adhesion, may contribute to the increased accumulation of mature B cells in the LTβR^-/-^ BM. While LTβR signaling is known to regulate immune cell migration by controlling adhesion molecule and chemokine expression in SLOs (15, 123–125) and the thymus (26, 126, 127), its role in shaping the BM microenvironment and guiding B-cell chemotaxis remains poorly understood. LTβR signaling, particularly via LTα_1_β_2_ engagement, is essential for T-cell progenitor homing to the thymus (128–130), the organization of its medullary architecture (26, 27, 131), and the egress of mature T cells, which relies on a specific LTβR-dependent endothelial subset known as portal endothelial cells (27, 130, 132). Given this role in thymocyte egress, it is conceivable that LTβR signaling similarly facilitates egress of mature B cell from the BM.

While these results warrant further investigation of the BM microenvironment, the increase in B cell frequency/number is not restricted to the BM but is also present in the spleen (25) and, as we have shown here, in the PerC of uninfected LTβR^-/-^ mice. As with BM B cells, alterations in the local microenvironment and/or chemotaxis may contribute to this phenotype. In addition, the absence of SLOs in LTβR^-/-^ mice, including LNs and gut-associated lymphatic tissue (GALT) (11), may promote the accumulation of mature B cells in alternative compartments such as the BM and the PerC.

In the PerC of uninfected LTβR^-/-^ mice, B-1a cells were a notable exception to the otherwise increased B-cell frequencies compared to WT mice. In our previous study, a host-pathogen network prediction model based on RNASeq data from murine lung tissue following *T. gondii* infection suggested that LTβR deficiency could upregulate the “B-cell receptor signaling pathway” gene set (25). If altered BCR signaling contributes to the observed B-cell phenotype in LTβR^-/-^ mice, the relative unresponsiveness of peritoneal B-1 cells (93, 133–135) - particularly B-1a cells (136, 137) - to BCR stimulation, compared to B-2 cells, may explain why B-1a cells remained unaffected despite the overall increase in B-cell frequencies in LTβR^-/-^ mice. Notably, although B-1a cell frequencies were comparable to those of WT mice, their absolute numbers were elevated due to an expanded leukocyte compartment in the LTβR^-/-^ PerC.

In addition to the B-cell compartment, we also observed changes in the T-cell compartment in the BM of uninfected LTβR^-/-^ mice. In contrast to BM B cells, BM T cells (CD3e^+^) were not substantially increased in LTβR^-/-^ mice, despite their shared capability to express LTβR ligands, their overlapping BM niches (122), and their similarly increased presence in PB and tissue infiltrations in LTβR^-/-^ mice (11). However, the significant shift in subset frequencies, characterized by a decrease in DN T cells and an increase in CD4^+^ and CD8^+^ T cells, indicates that LTβR deficiency does impact the BM T-cell compartment. While DN T cells are enriched in the BM (77), their origin remains rather unclear, with evidence supporting both thymus-dependent and thymus-independent pathways, including differentiation from both CD4^+^ and CD8^+^ T cells (78, 138). The absence of LTβR signaling may disrupt DN T-cell development or migration, leading to their reduced frequencies in the BM. Notably, uninfected LTβR^-/-^ mice exhibit increased numbers of CD4^+^ (and CD8^+^) T cells in the thymus (27, 132), and CD4^+^ regulatory T cells (T_regs_) are particularly dependent on LTα_1_β_2_-LTβR interactions for efficient transendothelial migration (139, 140). Our data, therefore, clearly show that the absence of LTβR signaling also affects the BM T-cell compartment.

During inflammation, the BM microenvironment is disrupted, with lymphopoiesis suppressed in favor of emergency granulopoiesis to replenish the depleted granulocyte compartment for host defense (141–143). While uninfected LTβR^-/-^ mice had significantly higher B-cell numbers and frequencies in the BM compared to WT mice, *T. gondii* infection led to a marked reduction in early-stage B-cell subsets in both genotypes. By contrast, later-stage subsets, particularly mature B cells, were significantly more resistant to inflammation-induced reduction compared to their WT counterparts. Similar reductions in early-stage B cells have been reported during infections with other protozoan parasites, such as *Trypanosoma brucei* (144) and *Plasmodium chabaudi* (145); however, mature BM B cells were not assessed in these studies. The reduction of early-stage B cells has been linked to the chemokine CXCL12, which is essential for B-cell development and regulates their retention in the BM through the very late antigen 4 (VLA-4/integrin α_4_β_1_) and vascular cell adhesion protein 1 (VCAM-1) axis (146–150). Proinflammatory cytokines, particularly TNFα and IL-1β, downregulate stromal CXCL12 expression, promoting the egress of lymphocytes, including early-stage B cells, into the blood (107). Notably, while both LTβR^-/-^ and WT mice had reduced CXCL12 concentrations in their BM during infection, we did not detect a significant increase in early-stage B cells in the PB on day 9 p.i. Given their exceptional sensitivity to apoptosis (151) and the advanced stage of early B-cell depletion, it is conceivable that most mobilized early-stage B cells were cleared from the circulation by the time of analysis on day 9 p.i.

During B-cell development, sensitivity to CXCL12-guided chemoattraction gradually declines as CXCR4 expression decreases, allowing later-stage B cells to exit the BM (147, 150). However, unlike circulating B cells, mature BM B cells still respond to sustained CXCL12-induced adhesion, albeit to a lesser extent than earlier B-cell subsets (147). Blocking CXCL12 signaling via CXCR4 depletion in CD19^+^ BM B cells increases their localization to BM sinusoids rather than the parenchyma and significantly reduces mature B-cell numbers in the BM (149). However, this reduction may, in part, result from disrupted MIF signaling, as MIF is a critical survival factor for mature B cells in the BM (152, 153), acting through CXCR4 (and CXCR2) (154, 155). In addition, B-cell homing to the BM is impaired in mice with reduced CXCL12 expression (107). Collectively, these findings indicate that CXCL12 remains important for mature B-cell retention and localization within the BM, even if their responsiveness to its signals diminishes over time. Therefore, while the CXCL12-dependent reduction of early and mature B cells in WT BM during inflammation aligns with previous findings, mature BM B cells in infected LTβR^-/-^ mice remained unaffected despite CXCL12 reduction, suggesting retention through altered or unknown mechanisms during systemic inflammation. Notably, while our BM transcriptome data revealed genotype-dependent differences in the expression of certain migration- and adhesion-related genes, no substantial differences were observed in the expression of mRNAs of typical LTβR-inducible adhesion molecules, such as *Vcam1*, intercellular adhesion molecule 1 (*Icam1*), *Sele* (E-selectin), and mucosal vascular addressin cell adhesion molecule 1 (*Madcam1*) (10, 20, 125, 127, 156), either between genotypes or during *T. gondii* infection. The regulation of these adhesion molecules in the BM in the absence of LTβR signaling requires further investigation.

Furthermore, BMPCs (TACI^+^ CD138^+^) were comparable between genotypes or even increased in LTβR^-/-^ mice on day 9 p.i., despite the absence of LNs and GALT and defective splenic GC formation in these mice (11, 13, 17). Alternative PC differentiation in the BM, independent of GC maturation or T-cell help (120, 157, 158), and/or B-1 cell-derived BMPCs (CD138^+^), a key source of natural IgM (97, 115), could account for the observed IgM^+^ BMPCs in LTβR^-/-^ mice. The marked reduction of IgA^+^ BMPCs aligns with reports of impaired IgA production in LTβR^-/-^ mice (13, 123, 159). IgA production primarily occurs in the GALT, including PPs (160), mesenteric lymph nodes (MLNs) (161), and the lamina propria (LP) (162); however, PPs and MLNs are absent in LTβR^-/-^ mice (11), and recruitment of B cells into the LP is dependent on LTβR expression of LP stromal cells (123). Overall, LTβR signaling appears crucial for IgA^+^ PC generation, prior to and after pathogen infection.

*T. gondii* infection also affected the PerC B-cell compartment, which contains both B-1 cells and conventional B-2 cells. While the frequencies of both subsets were markedly reduced on day 9 p.i. in both genotypes, an earlier reduction of B-1 cells was observed on day 3 p.i., whereas B-2 cells remained unchanged at this point. Peritoneal B-1 cell reduction has been reported following i.p. infections, with kinetics ranging from as early as 3 hours (after *Escherichia coli* injection, associated with increased B-1 cell egress [117]) to up to 15 days (after *Trypanosoma cruzi* infection, linked to B-1 cell differentiation into peritoneal PCs rather than increased apoptosis [163]). Given the early onset of B-1 cell reduction, we hypothesize that B-1 cell egress with kinetics specific to an intraperitoneal *T. gondii* infection accounts for this decline. CXCL13, which mediates peritoneal B-1 cell migration (116, 117), showed no genotype-specific differences in the serum or BM^SN^. Notably, while CXCL13 production in SLOs is LTα_1_β_2_-dependent and primarily derived from follicular stromal cells and FDCs (13, 15, 124, 164–166), macrophages serve as major producers of CXCL13 in the PerC, independent of LTα_1_β_2_-LTβR signaling (116, 117, 167, 168). While we hypothesize functional CXCL13-induced B-1 cell egress from the PerC in LTβR^-/-^ mice, their fate remains unknown due to the severe alteration or absence of SLOs in these mice.

In addition, the LTβR^-/-^ PerC contained higher leukocyte numbers even before *T. gondii* infection, suggesting an elevated basal immune activation status, consistent with previous reports of tissue infiltration by CD4^+^ T cells and B cells, as well as autoantibody production in LTβR^-/-^ mice under steady-state conditions (11, 13, 25, 118). During *T. gondii* infection, LTβR^-/-^ PerC leukocyte numbers remained significantly elevated compared to the WT PerC. Necropsy revealed moderate to marked perivascular inflammatory infiltrates in the lungs and livers of LTβR^-/-^ mice, which were less pronounced in WT animals. These results indicate altered lymphocyte migration, as discussed earlier, or differences in infection dynamics and immune responses between genotypes.

Furthermore, on day 9 p.i., the LTβR^-/-^ PerC immune cell composition was dominated by neutrophils instead of T cells and showed increased cell death, both in contrast to WT PerC. LTβR signaling has been shown to regulate neutrophil metabolism and prevent excessive inflammation (3). While we did not assess their functionality, the accumulation of neutrophils in the LTβR^-/-^ PerC, along with increased cell death, may indicate exacerbated peritoneal inflammation and potential immune pathology in these mice.

Overall, the BM transcriptome data indicate that although the immune response, particularly IFN-related genes, was upregulated during *T. gondii* infection in LTβR^-/-^ BM, it remained markedly diminished compared to the WT BM. This includes significantly lower expression of multiple genes associated with MHCII and antigen presentation, which aligns with our finding of reduced MHCII surface expression on proinflammatory (Ly6C^+^) monocytes/macrophages in LTβR^-/-^ BM. During *T. gondii* infection, Ly6C^+^ monocytes and their precursors are primed with IFNγ from activated NK cells before BM egress, promoting MHCII upregulation (169, 170). Given the near absence of NK cells in LTβR^-/-^ mice (13, 24), including in the BM (as shown in this study), the impaired monocyte activation likely reflects a disrupted IFNγ-driven priming process. This also aligns with previous reports of a significantly delayed systemic IFNγ response in LTβR^-/-^ compared to WT mice (25, 29). Notably, while IFN-related gene set enrichment persisted in LTβR^-/-^ BM throughout the infection, its decline from day 6 to day 9 p.i. in WT BM suggests immune downregulation, likely reflecting emerging parasite control and the need to prevent excessive immunopathology.

In addition, the LTβR^-/-^ BM showed a consistent enrichment of the "TNFα signaling via NF-κB" gene set across all investigated time points. Since this enrichment was already present in uninfected mice, it is unlikely to be driven by *T. gondii* infection but may instead represent a compensatory mechanism attempting to counterbalance the absence of LTβR signaling (171). Given that the LTβR and TNF receptors (TNFR1/TNFRp55 and TNFR2/TNFRp75) share downstream signaling components of the classical NF-κB pathway (172–174), activation of these could explain the observed enrichment. However, as these findings are based on bulk RNA sequencing, it remains unclear whether this enrichment occurs broadly across cell populations or is restricted to specific TNF-responsive cells, and the underlying mechanism(s) require further investigation.

In conclusion, our study identifies distinct and novel alterations in immune cell (sub)populations in LTβR^-/-^ mice before and during *T. gondii* infection. This includes elevated frequencies of mature B cells in the BM and PerC, unaltered B-1a cell frequencies in the PerC, and reduced DN T cell frequencies in the BM. While LTβR signaling was previously known to influence T-cell migration and development in the thymus, the results from this study strongly suggest that LTβR signaling also plays an important role in the homeostasis and/or migration of mature B cells in the BM and PerC. In addition, our study provides valuable insights into the BM transcriptome in the absence of LTβR expression during protozoan parasite infection, thereby contributing to a deeper understanding of the complex and multifaceted roles of LTβR signaling in immune responses.

## MATERIALS AND METHODS

### Mice

LTβR^-/-^ mice were described previously (11) and back-crossed to a C57BL6/N background for at least 10 generations. Wild-type (WT) littermates were used as controls. Mice were housed in the animal facility of the Heinrich Heine University Düsseldorf under specific-pathogen-free (SPF) conditions and were 8 to 16 weeks old at the time of infection. CD1 mice from Charles River Breeding Laboratories were used to maintain and propagate ME49 *Toxoplasma gondii* for infection experiments. All animal experiments were conducted in strict accordance with the German Animal Welfare Act. The protocols were approved by the local authorities (Permit no. 81-02.04.2018.A406, 81-02.04.2021.A060, 81-02.05.40.18.082, and 81-02.04.40.23.VG055). All applicable international, national, and institutional guidelines for the care and use of animals were followed.

### *T. gondii* cyst preparation and infection experiments

ME49 (type II strain) cysts were isolated from the brain of CD1 mice 11 to 19 weeks after infection via Ficoll-Paque gradient centrifugation. Briefly, the murine cerebrum was homogenized by passaging through successively thinner cannulas (smallest size: 23G). After centrifugation (130 × *g*, 5 min, room temperature [RT]), the pellet was resuspended in 20 mL PBS. 10 mL of Ficoll-Paque Plus (GE Healthcare, USA) was carefully layered below the PBS, followed by centrifugation (1,250 × *g*, 25 min, RT) without brakes. Pelleted cysts were washed with PBS, counted (10 cysts per mouse), and lysed with Trypsin-EDTA. Lysis was stopped with the addition of heat-inactivated (56°C, 30 min) fetal calf serum (FCS, Pan Biotech, Germany). After a final wash with PBS and centrifugation (660 × *g*, 10 min, RT), ME49 bradyzoites were resuspended in 0.2 mL PBS per murine recipient. Mice were infected via intraperitoneal injection and weighed and scored daily for the duration of the experiment.

### Mouse material preparation and processing

On day 3, 6, and 9 p.i., mice were anesthetized with 100 mg/kg ketamine (Zoetis, USA) and 10 mg/kg xylazine (Elanco, USA). *Peritoneal lavage* was performed by injection (22G) of 5 mL ice-cold PBS (+2% FCS, vol/vol) into the peritoneal cavity, followed by a gentle massage and extraction. Aliquots were prepared for flow cytometry and quantitative real-time PCR (qRT-PCR). *PB/serum*: mice were bled through the *vena cava inferior* (20G). For flow cytometry, 200 µL of PB was immediately added to 50 µL 0.5 M EDTA and stored on ice. After washing with 25 mL PBS (+2% FCS, vol/vol) and centrifugation (470 × *g*, 7 min, 4°C), red blood cells (RBC) were lysed with Erylysis buffer (Morphisto, Germany). Leukocytes were then counted and used for flow cytometry surface staining. Remaining blood was allowed to coagulate for 30 min at room temperature (RT) followed by two centrifugation steps (10 min, 8,000 × *g*) to generate serum. *Bone marrow*: both murine femora were dissected, and BM cells were isolated and pooled via centrifugation. Briefly, femora were cut open at the knee joint side and placed in punctured 0.5 mL tubes that were then placed in 1.5 mL tubes. After centrifugation (12,100 × *g*, 15 sec, RT), the BM supernatant was removed for cytokine measurement. After RBC lysis, remaining leukocytes were counted, and aliquots were prepared for flow cytometry, qRT-PCR, and RNA sequencing.

### Cell surface staining for flow cytometry

Cells were stained in U-bottom polystyrene plates and were incubated with Fc-blocking CD16/32 antibody (1:100) in staining buffer (1× PBS with 2 mM EDTA and 2% FCS [vol/vol]) for 30 min at 4°C. Fluorophore-labeled antibodies were prepared in staining buffer, added to cells (final volume: 50 µL), and incubated for 30 min (4°C, light-protected). Cells were then washed, preserved by the addition of 4% paraformaldehyde for 15 min at RT, and analyzed with a BD LSRFortessa II flow cytometer and FlowJo v10.8 software. All antibodies and dyes are listed in Table S1, all identified immune cell populations are listed in Table S2, and all gating strategies are shown in Fig. S1, S5, S6, and S8.

### Detection of parasite burden

Bone marrow and peritoneal lavage samples were centrifuged (10,000 × *g*, 5 min, RT), total DNA was isolated from pellets using a DNA isolation kit (Genekam, Germany) according to the manual, and samples were set to 100 ng/µL DNA. TgB1 primer (Forward: 5′-GCT AAA GGC GTC ATT GCT GTT-3′, Reverse: 5′-GGC GGA ACC AAC GGA AAT-3′) and a FAM-probe (5′-FAM-ATC GCA ACG GAG TTC TTC CCA GAC GT-BHQ1-3′) were purchased from Metabion (Germany) to amplify and detect a defined sequence of the 35-fold repetitive B1 gene from *T. gondii*, qRT-PCR was performed on Bio-Rad CFX-96 systems. DNA isolated from a defined number of ME49 tachyzoites (2914 ± 214 /µL) was used to generate a standard curve and calculate parasite loads.

### Cytokine measurement

Murine cytokines BAFF, CXCL12, and CXCL13 were measured with a custom-selected bead-based multiplex panel (LEGENDplex from BioLegend, USA). BM^SN^ was used undiluted. Samples were acquired with a BD FACSCanto II or LSRFortessa II and analyzed with Qognit software (BioLegend).

### Histology

Liver, lung, spleen, kidney, brain, and femora were harvested from WT and LTβR^-/-^ mice, fixed in 4% neutral buffered formalin for 48 hours, and stored in 70% ethanol at 4°C until further processing. 5 µm sections were cut, transferred onto glass slides, and stained with a standard hematoxylin/eosin protocol. Femur samples were decalcified in RDO solution (Apex Engineering, USA) for 1 hour before processing.

### Generation of QuantSeq 3′ mRNA-sequencing data

Bone marrow samples of uninfected (d0) and *T. gondii*-infected (ME49, bradyzoites from 10 cysts, i.p.) WT and LTβR^-/-^ mice were generated as described and stabilized in RNAprotect Tissue Reagent (Qiagen, Germany). Total RNA was isolated using the RNeasy Plus Mini Kit (Qiagen, Germany), quantified (Qubit RNA HS Assay, Thermo Fisher Scientific, USA), and quality measured by capillary electrophoresis using the Fragment Analyzer and the “Total RNA Standard Sensitivity Assay” (Agilent Technologies, USA). All samples in this study showed high-quality RNA Quality Numbers (RQN mean = 9.8). Library preparation was performed according to the manufacturer’s protocol using the Lexogen QuantSeq 3′ mRNA-seq Library Prep Kit FWD with UMI’s (Lexogen, Austria). The input amount was 25 ng total RNA. Bead-purified libraries were normalized and finally sequenced on the NextSeq2000 system (Illumina, USA) with a read setup of 1 × 100 bp. The BCL Convert Tool (version 3.8.4) was used to convert the bcl files to fastq files as well as for adapter trimming and demultiplexing.

### Analysis of the mRNA-sequencing data

Sequencing data were processed following the recommended protocol on the Lexogen website (https://faqs.lexogen.com/faq/quantseq-with-udi-v2). Using the umi_tools software package (version 1.1.4), we extracted the Unique molecular identifier (UMI) from the reads of the FASTQ files and removed the adjacent TATA spacer by the “umi_tools extract” command, resulting in FASTQ files with the UMI in the read names (175). Since QuantSeq 3′ mRNA-seq reads contain part of the poly-A tail, we next trimmed any sequenced poly-A sequences and the Lexogen adapter sequences from the read ends, as well as low-quality bases by cutadapt (version 3.5) (176). Reads potentially containing rRNA transcripts were removed using the sortMeRNA tool (version 4.3.7), using the recommended latest database “smr_v4.3_default_db” (177). Data quality was assessed at every step using the tools FASTQC and Multiqc.

For reads alignment, we generated a fused meta-genome with the STAR alignment tool (version 2.7.10 a) (178), combining the genomic sequences and genome annotations of the mouse (GRCm39 ensembl version 111) and toxoplasma (ensemble protists version 55) reference from Ensembl. The reads were then aligned to the fused STAR genome index, which was generated setting the minimal splice site overlap to 99 (--sjdbOverhang 99). Uniquely mapped reads were selected from the BAM files using the samtools software package (version 1.13) (179). Duplicated reads were then removed from the BAM files with umi_tools. Finally, gene count matrices were generated from the BAM files by the featureCount software (version 2.0.3) (180). Differential gene expression analysis was performed on the gene count matrices by a custom R script, using the DESeq2 R package (version 1.44.0) (181). The results of the differential gene expression analysis were then used for GSEA, using the fgsea R package (version 1.30.0) (182).

### Statistical analysis

GraphPad Prism software (version 10) was used for data analysis. Symbols represent individual animals, columns represent mean values, and error bars represent the ±standard deviation (SD). Outliers were identified and excluded from the data using the ROUT test (Q = 1%), after which the Shapiro-Wilk test of normality was performed. Parametric data were analyzed using two-way analysis of variance (ANOVA) corrected for multiple comparison using Tukey’s *post hoc* test. Nonparametric data were analyzed using the Kruskal-Wallis test followed by Dunn’s multiple comparison test.

## Data Availability

Raw sequencing data were submitted to the ENA database under accession number PRJNA1177674. Code that was used to analyze the data can be found on github at https://github.com/caggtaagtat/QuantSeq-3-mRNA-sequencing.

## SUPPLEMENTAL MATERIAL

Fig. S1 to S4 iai.00408-25-s0001.pdf BM B cell gating strategy (S1), absolute BM B cell numbers (S2), peripheral blood (PB) B cells (S3) and BM plasma cells (BMPCs) (S4).

Fig. S5 to S8 iai.00408-25-s0002.pdf BM T cell gating strategy (S5), BM pDC, NK cell and myeloid cells gating strategy (S6), absolute numbers of non-B cells in the BM, PB and PerC (S7) and PerC gating strategy (S8).

Fig. S9 and S10 iai.00408-25-s0003.pdf Complete GSEA of LTßR^-/-^ vs WT (S9) and intra-genotype comparisons (S10).

Fig. S11 to S13 iai.00408-25-s0004.pdf Absolute PerC B cell numbers and direct B-1a gating (S11), absolute PerC neutrophils and T cells (S12), and histology of kidney sections from WT and LTßR^-/-^ mice (S13).

Supplemental tables iai.00408-25-s0005.xlsx Table S1 and S2.

ASM does not own the copyrights to Supplemental Material that may be linked to, or accessed through, an article. The authors have granted ASM a non-exclusive, world-wide license to publish the Supplemental Material files. Please contact the corresponding author directly for reuse.
