# Supplementary material for: Lymphotoxin beta receptor-/- mice display altered B- and T-cell subpopulations in the bone marrow and peritoneal cavity after Toxoplasma gondii infection
Source: Infect Immun. 2025 Sep 9;93(10):e00408-25. doi: 10.1128/iai.00408-25 (PMC12519803; doi:10.1128/iai.00408-25)
Supplement: Fig. S1 to S4 — BM B cell gating strategy (S1), absolute BM B cell numbers (S2), peripheral blood (PB) B cells (S3) and BM plasma cells (BMPCs) (S4). [file iai.00408-25-s0001.pdf]

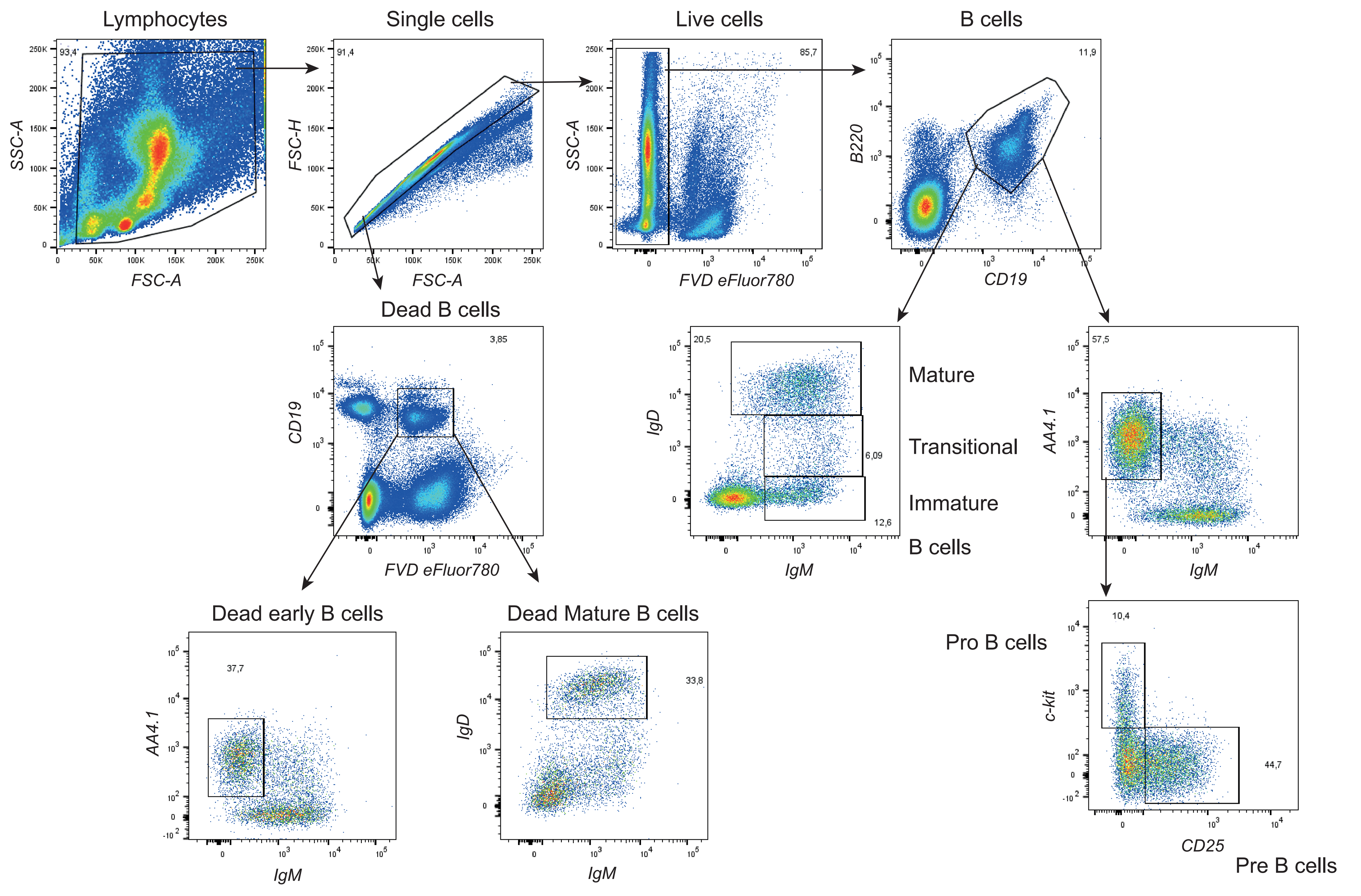

Fig. S1: Gating strategy for the detection of B cell subpopulations in the BM.

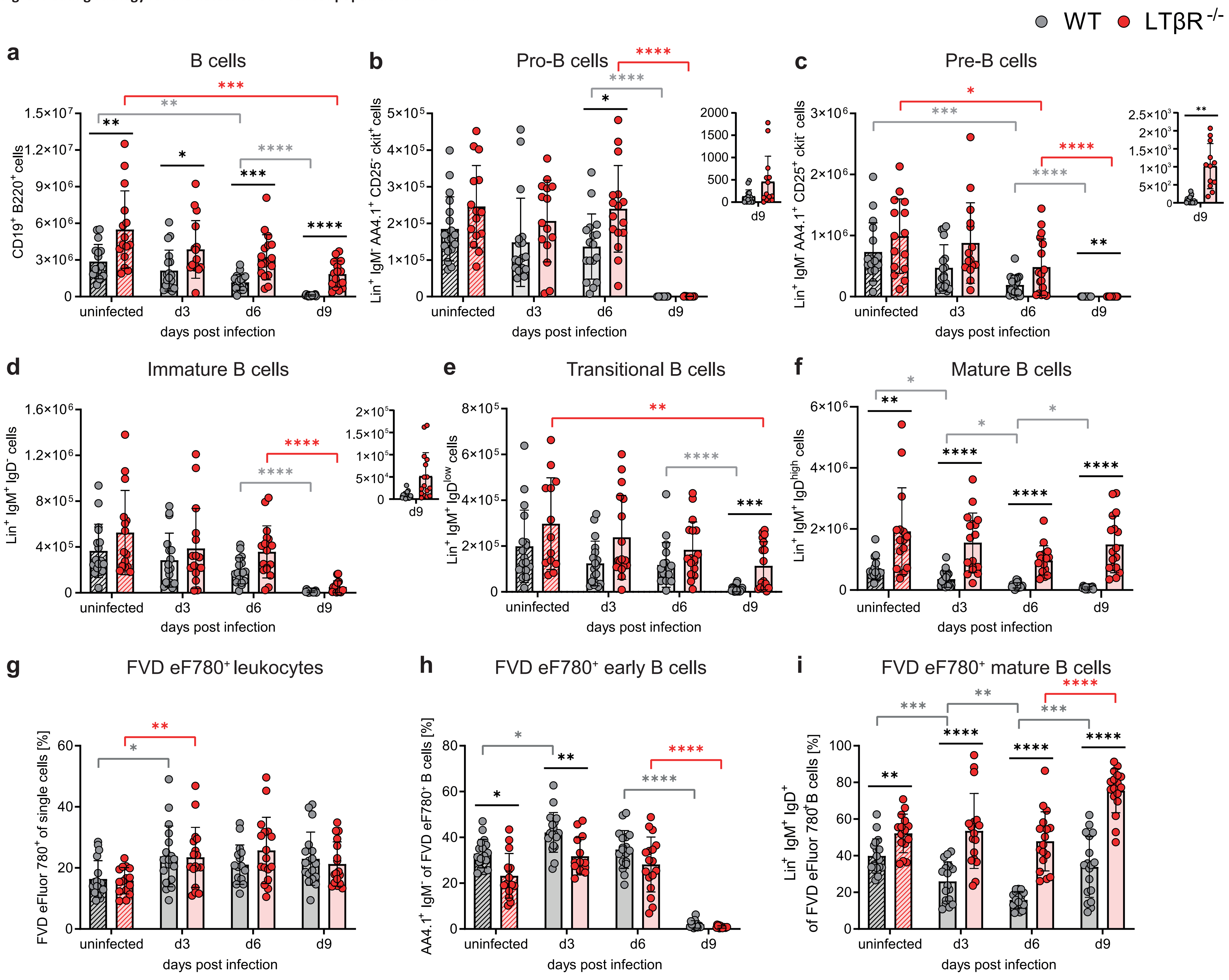

Fig. S2: Absolute numbers of B cell subpopulations and frequencies of dead B cell subpopulations in the BM of LTβR$^{-/-}$ and WT mice during *T. gondii* infection. Using surface marker staining and flow cytometry, the following immune cell populations (for gating strategy see Fig. S1) in the BM of WT (n ≥ 12/group) and LTβR$^{-/-}$ (n ≥ 11/group) mice were identified: **(a)** pan-B cells (CD19$^+$ B220$^+$), **(b)** Pro-B cells (Lin$^+$ IgM$^-$ AA4.1$^+$ CD25$^-$ ckit$^+$), **(c)** Pre-B cells (Lin$^+$ IgM$^-$ AA4.1$^+$ CD25$^+$ ckit$^-$), **(d)** Immature B cells (Lin$^+$ IgM$^+$ IgD$^-$), **(e)** Transitional B cells (Lin$^+$ IgM$^+$ IgD^{low}), **(f)** Mature B cells (Lin$^+$ IgM$^+$ IgD^{high}), **(g)** dead leukocytes (FVD eFluor 780$^+$, % of single cells), **(h)** dead early B cells (FVD eFluor 780$^+$ CD19$^+$ AA4.1$^+$ IgM$^-$, % of dead B cells), and **(i)** dead mature B cells (FVD eFluor 780$^+$ CD19$^+$ IgM$^+$ IgD$^+$, % of dead B cells). All data shown represent at least three independent experiments; symbols represent individual animals and columns represent mean values ± SD. Lin$^+$ = CD19$^+$ B220$^+$. *, P < 0.05; **, P < 0.01; ***, P < 0.001; ****, P < 0.0001.

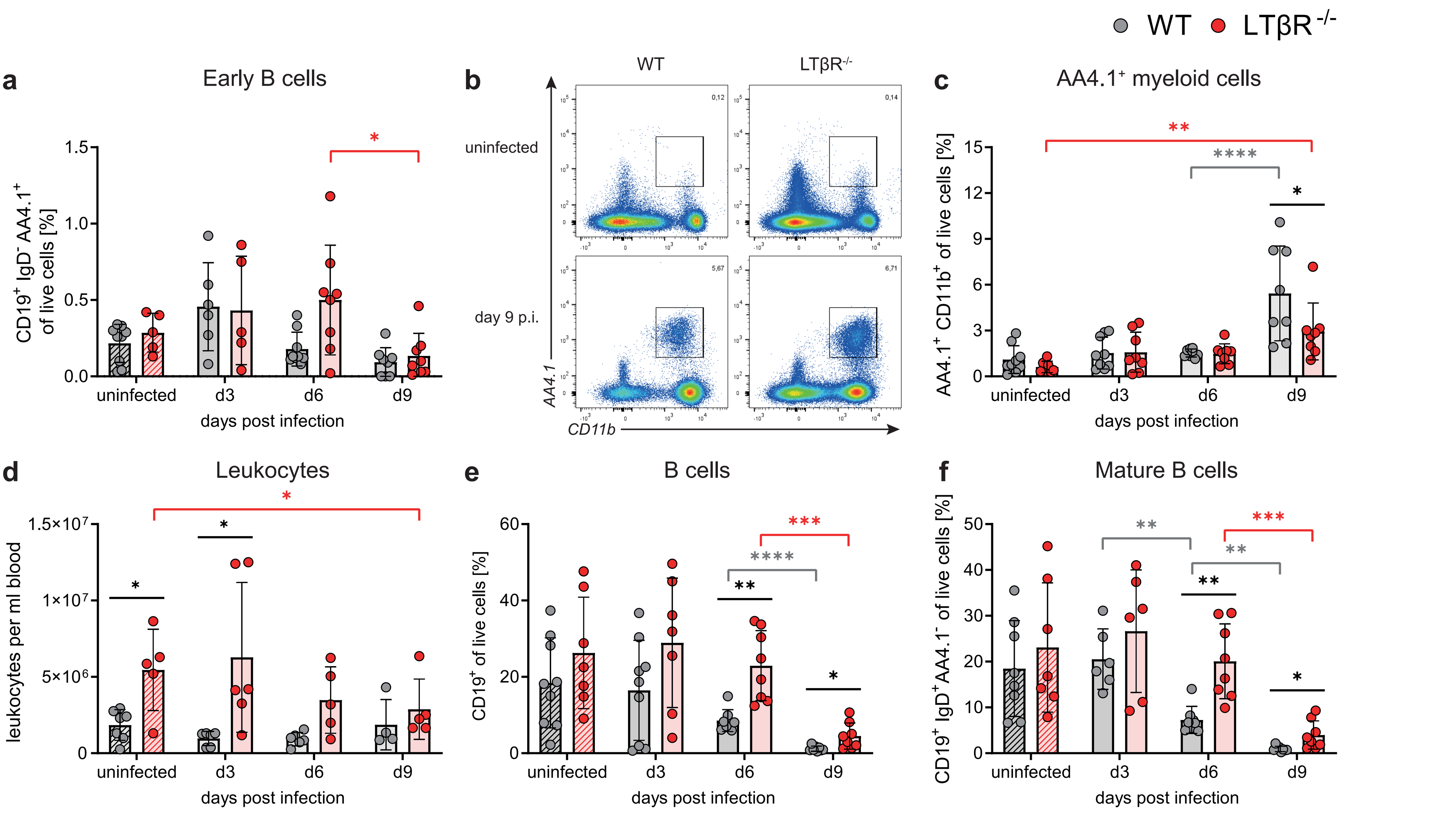

Fig. S3: Frequencies of B cell subpopulations and absolute leukocyte numbers in the PB of LTβR$^{-/-}$ and WT mice during *T. gondii* infection. Using surface marker staining and flow cytometry, the following immune cell populations in the PB of WT (n ≥ 6/group) and LTβR$^{-/-}$ (n ≥ 6/group) mice were identified and quantified as percentages of live cells: **(a)** early B cells (CD19$^+$ AA4.1$^+$ IgD$^-$), **(b & c)** AA4.1-positive myeloid cells (CD11b$^+$ AA4.1$^+$), (b) shows a set of representative images. **(d)** Absolute cell counts for PB leukocytes from WT (n ≥ 4/group) and LTβR$^{-/-}$ (n ≥ 5/group) mice. **(e)** Pan-B cells (CD19$^+$) and **(f)** Mature B cells (CD19$^+$ AA4.1$^-$ IgD$^+$). All data shown represent at least two independent experiments; symbols represent individual animals and columns represent mean values ± SD. *, P < 0.05; **, P < 0.01; ***, P < 0.001; ****, P < 0.0001.

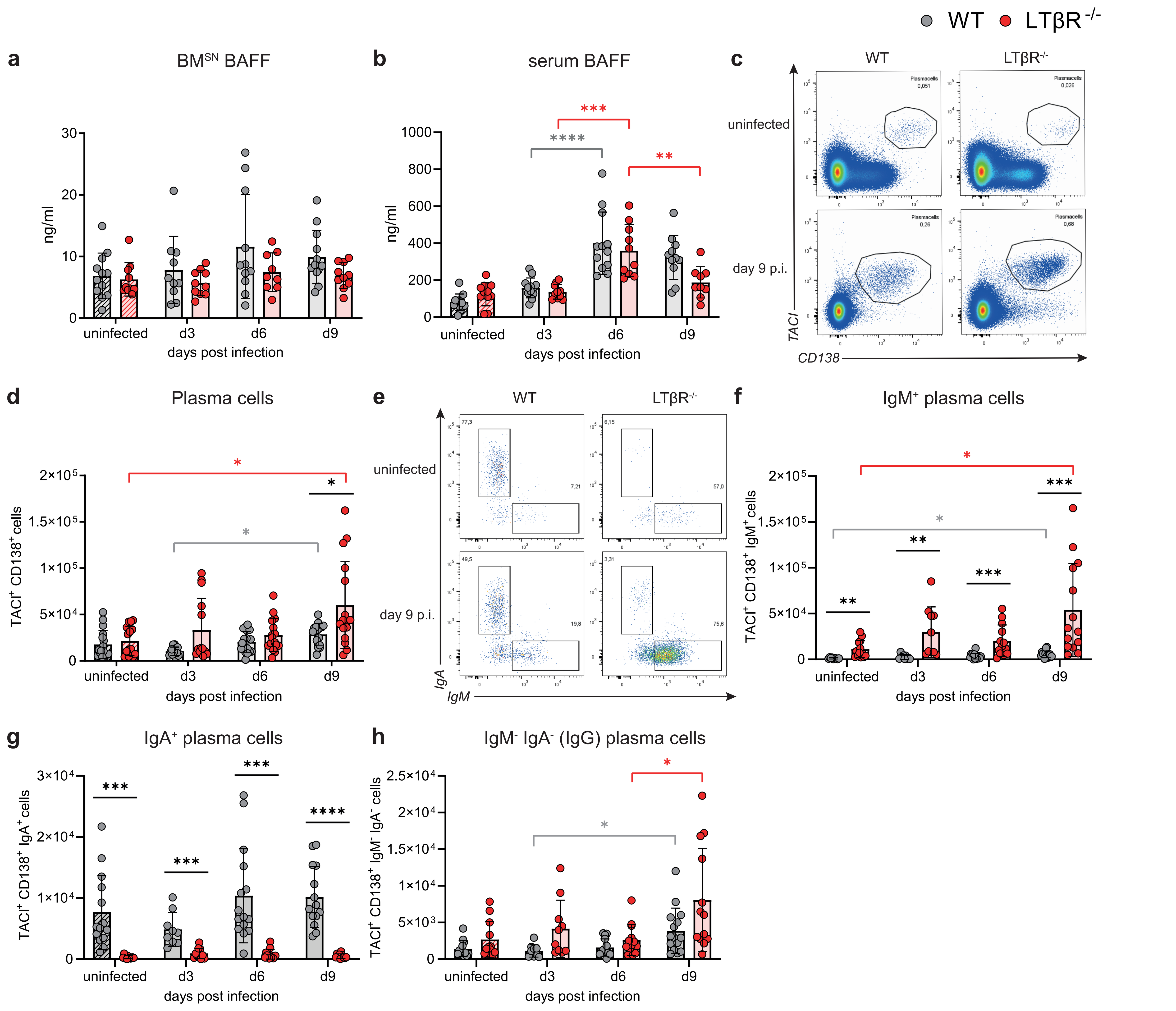

Fig. S4: BAFF in BM^{SN} and serum, and BM plasma cells of LTβR$^{-/-}$ and WT mice during *T. gondii* infection . BAFF measured in the **(a)** BM^{SN} and **(b)** serum of uninfected and infected WT (n ≥ 10/group) and LTβR$^{-/-}$ (n ≥ 8/group) mice via a bead-based immunoassay (LegendPlex, BioLegend, USA). Using surface marker staining and flow cytometry, the following immune cell populations in the BM of WT (n ≥ 9/group) and LTβR$^{-/-}$ (n ≥ 8/group) mice were identified: **(c)** Representative images of plasma cell (TACI$^+$ 138$^+$) gating, **(d)** absolute numbers of pan-plasma cells, **(e)** representative images of surface IgM and IgA plasma cell gating, **(f - h)** absolute numbers of (f) IgM plasma cells, (g) IgA plasma cells and (h) IgM$^-$ IgA$^-$ (IgG) plasma cells in the BM. BM^{SN} = bone marrow supernatant. Data shown represent at least three independent experiments; symbols represent individual animals and columns represent mean values ± SD. *, P < 0.05; **, P < 0.01; ***, P < 0.001; ****, P < 0.0001.
