# Supplementary material for: Lymphotoxin beta receptor-/- mice display altered B- and T-cell subpopulations in the bone marrow and peritoneal cavity after Toxoplasma gondii infection
Source: Infect Immun. 2025 Sep 9;93(10):e00408-25. doi: 10.1128/iai.00408-25 (PMC12519803; doi:10.1128/iai.00408-25)
Supplement: Fig. S5 to S8 — BM T cell gating strategy (S5), BM pDC, NK cell and myeloid cells gating strategy (S6), absolute numbers of non-B cells in the BM, PB and PerC (S7) and PerC gating strategy (S8). [file iai.00408-25-s0002.pdf]

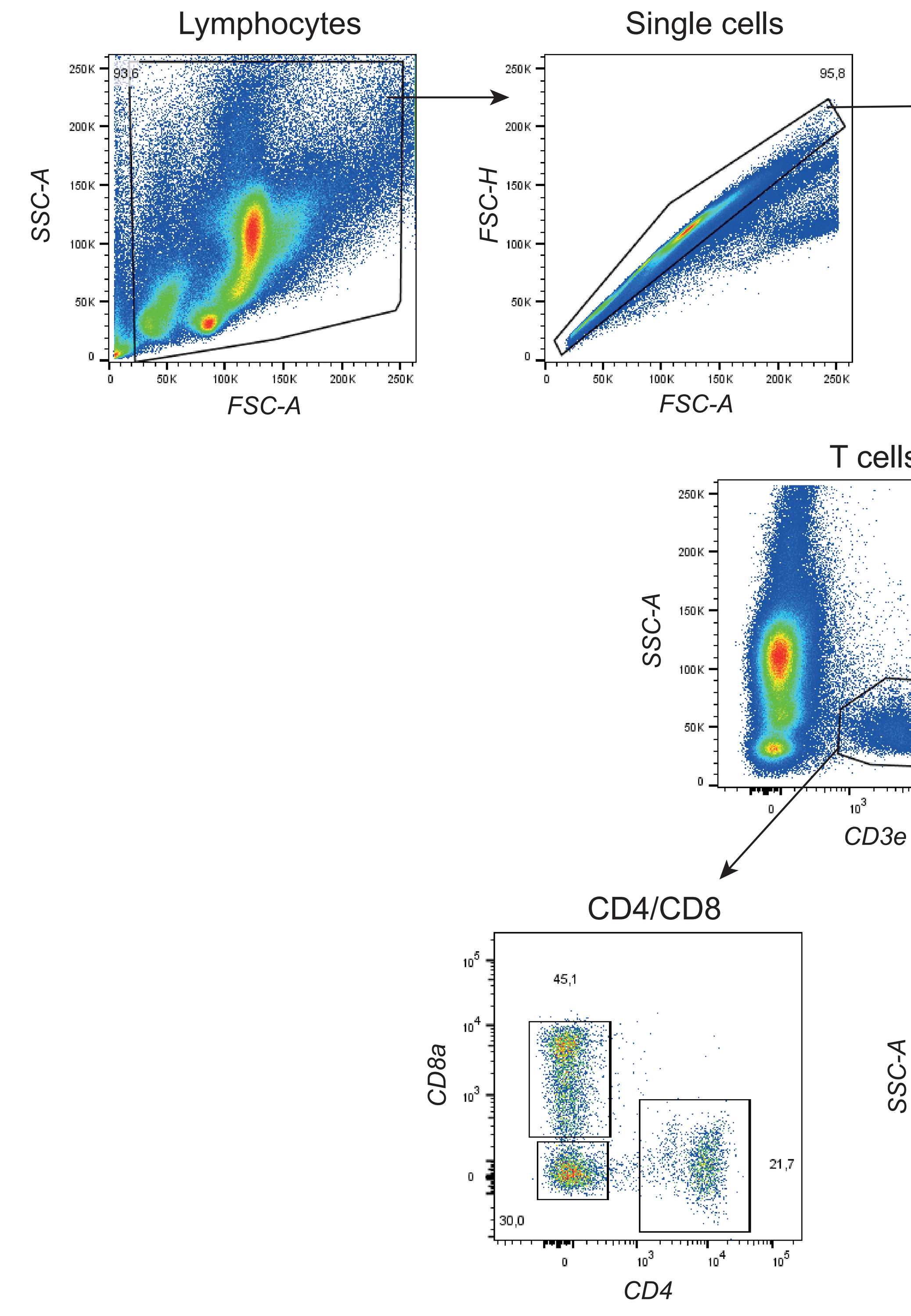

Fig. S5: Gating strategy for the detection of T cell subpopulations in the BM.

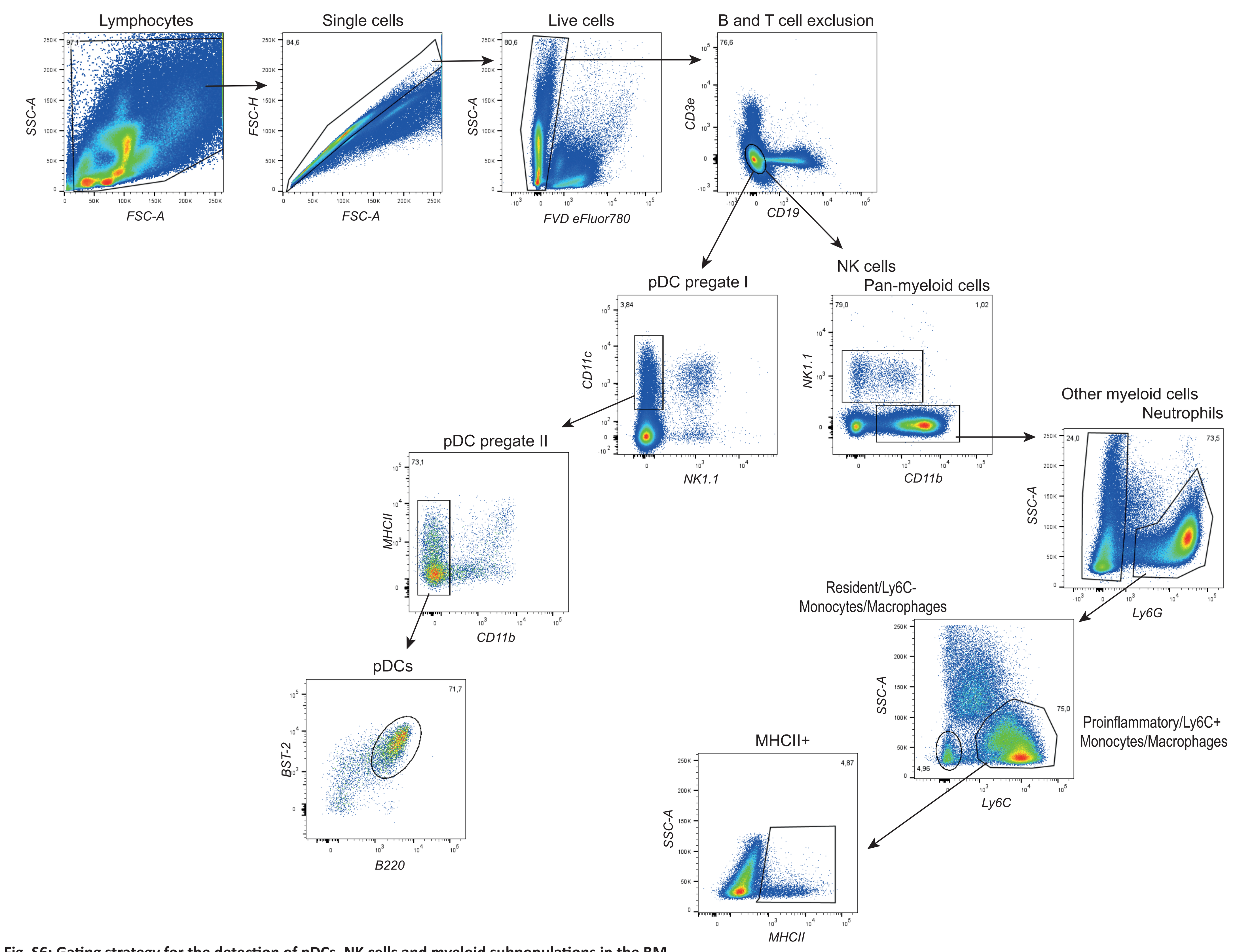

Fig. S6: Gating strategy for the detection of pDCs, NK cells and myeloid subpopulations in the BM.

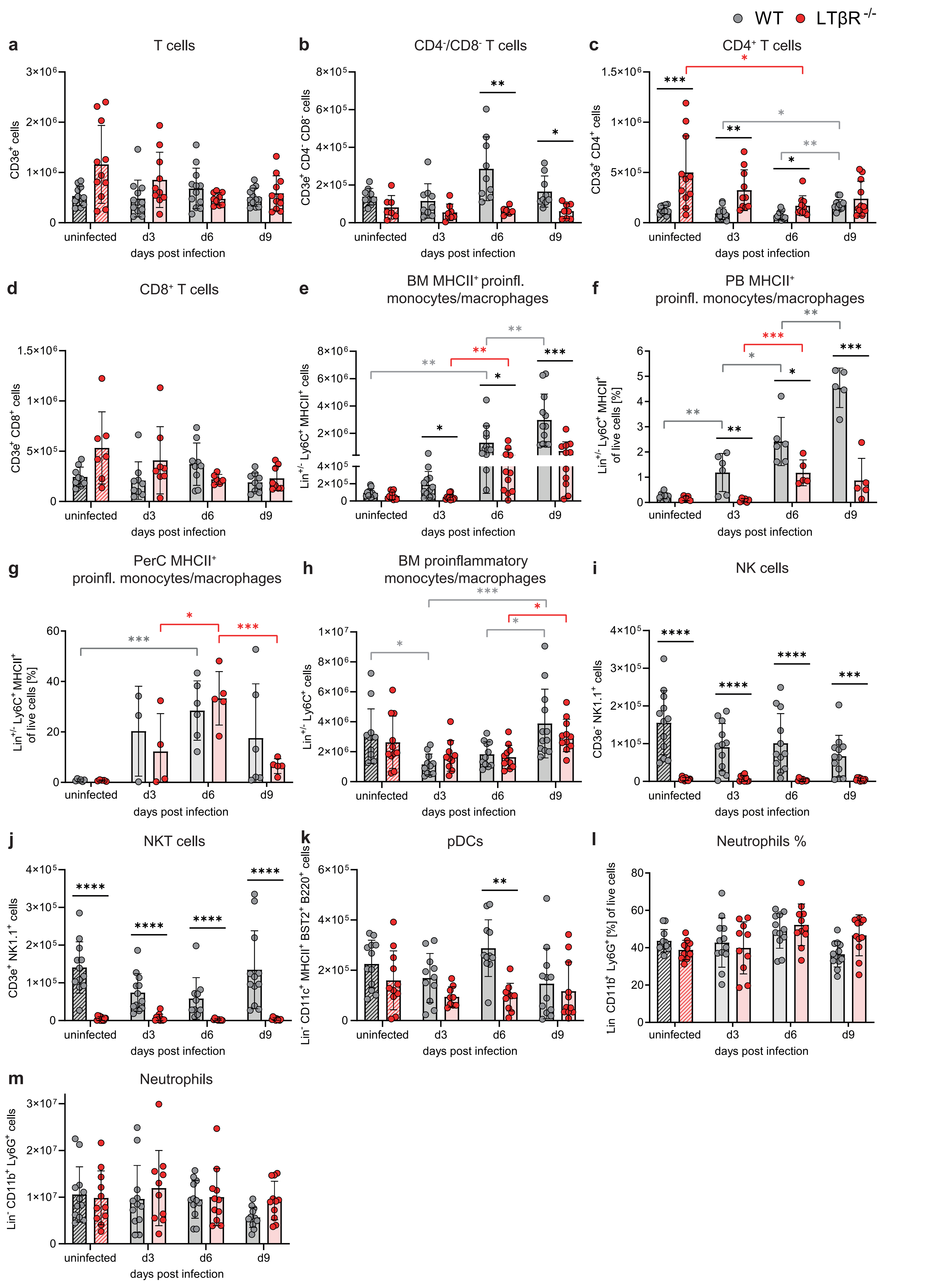

Fig. S7: Absolute numbers of BM T cells, (MHCII⁺) proinflammatory monocytes/macrophages, NK cells, NKT cells, pDCs, and neutrophils in the BM, and MHCII⁺ proinflammatory monocytes/macrophages in the PB and PerC. Using surface marker staining and flow cytometry, the following immune cell populations (for gating strategy see Fig. S5, S6 and S8) were identified: in the BM of WT (n ≥ 9/group) and LTβR$^{-/-}$ (n ≥ 7/group) mice: **(a)** pan-T cells (CD3e⁺), **(b)** double-negative T cells (CD3e⁺ CD4⁻ CD8⁻), **(c)** CD4⁺ T cells (CD3e⁺ CD4⁺), **(d)** CD8⁺ T cells (CD3e⁺ CD8⁺), and **(e)** MHCII-positive proinflammatory monocytes/macrophages (CD19⁻ CD3e⁻ NK1.1⁻ CD11b⁺ Ly6G⁻ Ly6C⁺ MHCII⁺). MHCII-positive proinflammatory monocytes/macrophages were also identified in the **(f)** PB and **(g)** PerC of WT and LTβR$^{-/-}$ (both n ≥ 5/group) mice, reported as % of live cells. Absolute numbers of BM **(h)** proinflammatory monocytes/macrophages (CD19⁻ CD3e⁻ NK1.1⁻ CD11b⁺ Ly6G⁻ Ly6C⁺), **(i)** NK cells (CD19⁻ CD3e⁻ NK1.1⁺), **(j)** NKT cells (CD3e⁺ NK1.1⁺) and **(k)** plasmacytoid DCs (pDCs; CD19⁻ CD3e⁻ NK1.1⁻ CD11c⁺ CD11b⁻ MHCII⁺ B220⁺ BST-2⁺). BM neutrophils (CD19⁻ CD3e⁻ NK1.1⁻ CD11b⁺ Ly6G⁺) reported as **(l)** frequency of live cells and **(m)** absolute numbers. BM = bone marrow. PB = peripheral blood. PerC = peritoneal cavity. Data shown represent: four independent experiments (BM) and two independent experiments each for PB and for PerC; symbols represent individual animals and columns represent mean values ± SD. *, P < 0.05; **, P < 0.01; ***, P < 0.001; ****, P < 0.0001.

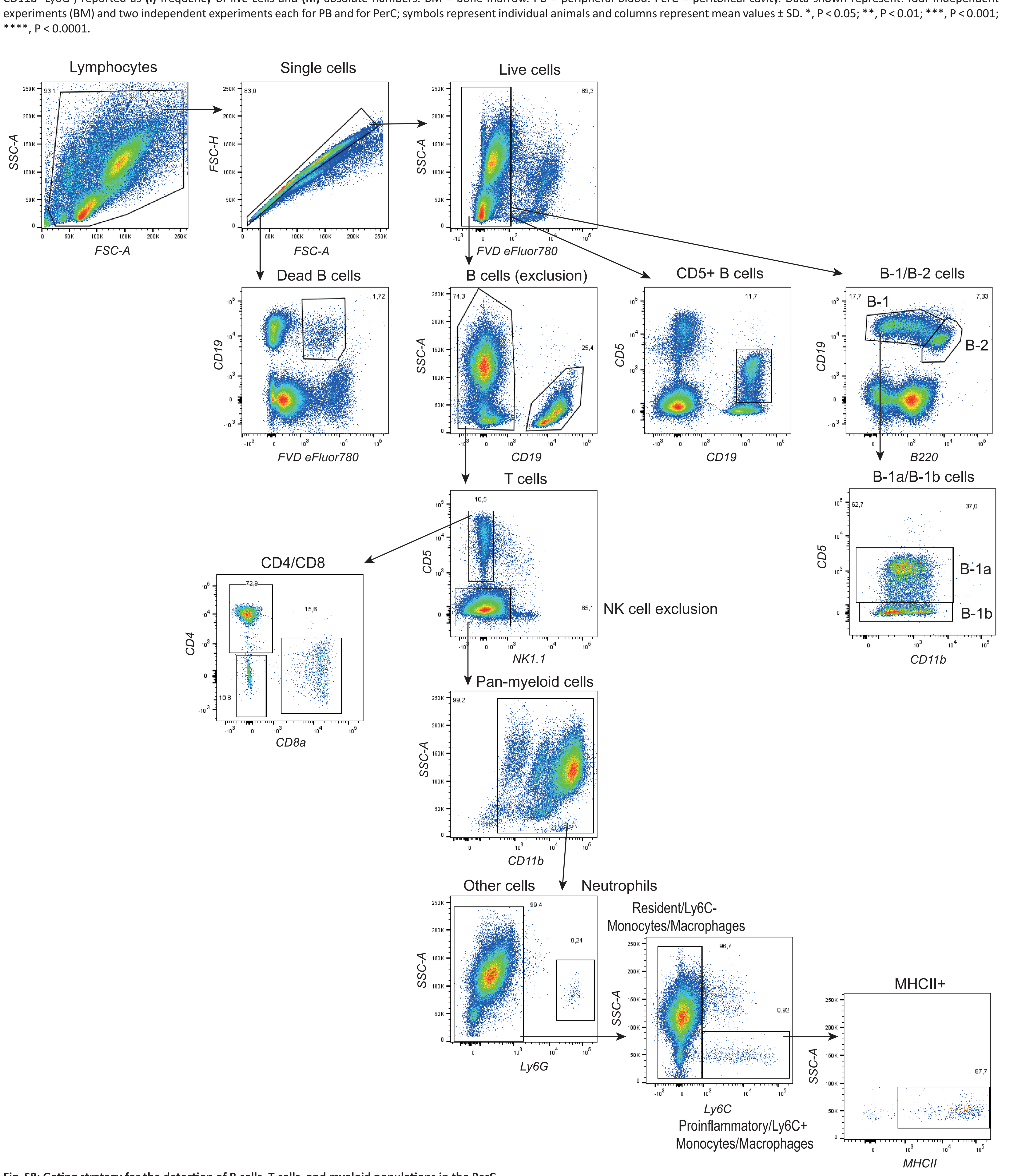

Fig. S8: Gating strategy for the detection of B cells, T cells, and myeloid populations in the PerC.
