# Supplementary material for: Lymphotoxin beta receptor-/- mice display altered B- and T-cell subpopulations in the bone marrow and peritoneal cavity after Toxoplasma gondii infection
Source: Infect Immun. 2025 Sep 9;93(10):e00408-25. doi: 10.1128/iai.00408-25 (PMC12519803; doi:10.1128/iai.00408-25)
Supplement: Fig. S9 and S10 — Complete GSEA of LTßR-/- vs WT (S9) and intra-genotype comparisons (S10). [file iai.00408-25-s0003.pdf]

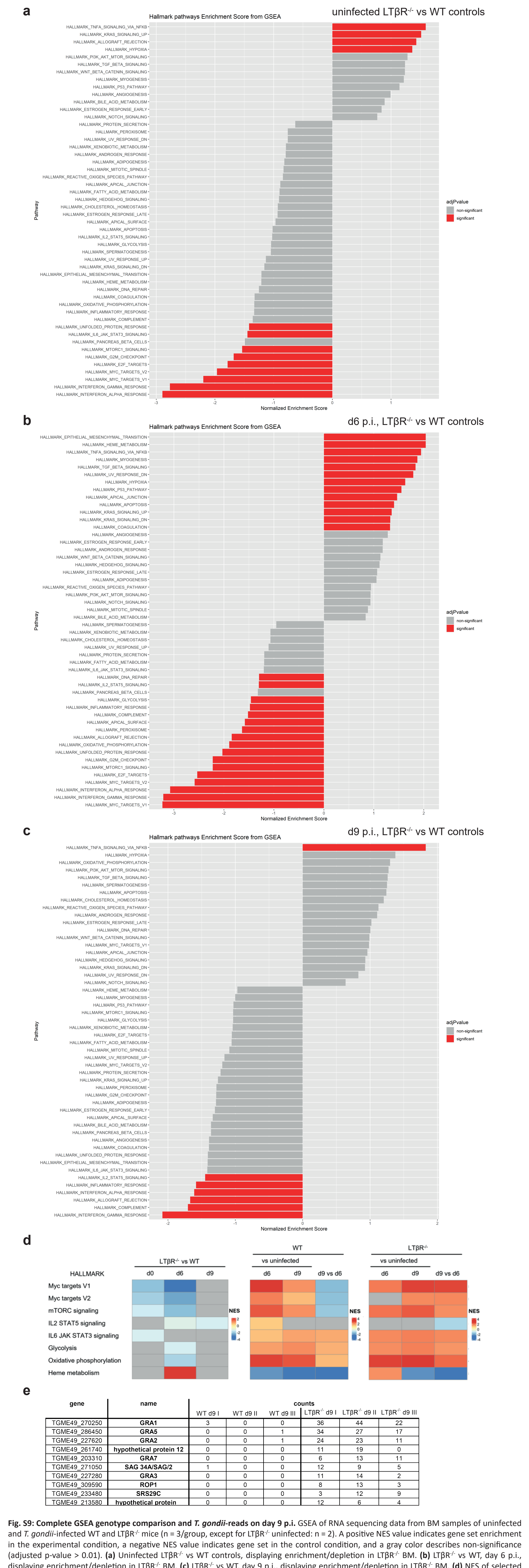

e

gene	name	counts					
		WT d9 I	WT d9 II	WT d9 III	LTβR-/- d9 I	LTβR-/- d9 II	LTβR-/- d9 III
TGME49_270250	**GRA1**	3	0	0	36	44	22
TGME49_286450	**GRA5**	0	0	1	34	27	17
TGME49_227620	**GRA2**	0	0	1	24	23	11
TGME49_261740	**hypothetical protein 12**	0	0	0	11	19	0
TGME49_203310	**GRA7**	0	0	0	6	13	11
TGME49_271050	**SAG 34A/SAG/2**	1	0	0	12	9	5
TGME49_227280	**GRA3**	0	0	0	11	14	2
TGME49_309590	**ROP1**	0	0	0	8	13	3
TGME49_233480	**SRS29C**	0	0	0	3	12	9
TGME49_213580	**hypothetical protein**	0	0	0	12	6	4

Fig. S9: Complete GSEA genotype comparison and *T. gondii*-reads on day 9 p.i. GSEA of RNA sequencing data from BM samples of uninfected and *T. gondii*-infected WT and LTβR$^{-/-}$ mice (n = 3/group, except for LTβR$^{-/-}$ uninfected: n = 2). A positive NES value indicates gene set enrichment in the experimental condition, a negative NES value indicates gene set in the control condition, and a gray color describes non-significance (adjusted p-value > 0.01). **(a)** Uninfected LTβR$^{-/-}$ vs WT controls, displaying enrichment/depletion in LTβR$^{-/-}$ BM. **(b)** LTβR$^{-/-}$ vs WT, day 6 p.i., displaying enrichment/depletion in LTβR$^{-/-}$ BM. **(c)** LTβR$^{-/-}$ vs WT, day 9 p.i., displaying enrichment/depletion in LTβR$^{-/-}$ BM. **(d)** NES of selected GSEA gene sets, summarized and represented as colors in a heat map. The left panel summarizes genotype comparisons (LTβR$^{-/-}$ vs. WT), displaying enrichment in LTβR$^{-/-}$ compared to WT controls. The middle panel summarizes intra-WT comparisons: d6 and 9 p.i. vs. uninfected controls, displaying enrichment in infected animals; and d9 vs. d6, displaying enrichment on day 9 p.i. compared to day 6 p.i. Similarly, the right panel summarizes intra-LTβR$^{-/-}$ comparisons: d6 and 9 p.i. vs. uninfected controls, displaying enrichment in infected animals; and d9 vs. d6, displaying enrichment on day 9 p.i. compared to day 6 p.i. **(e)** Absolute counts of *T. gondii*-derived transcripts found in the BM of infected WT and LTβR$^{-/-}$ mice on day 9 p.i.

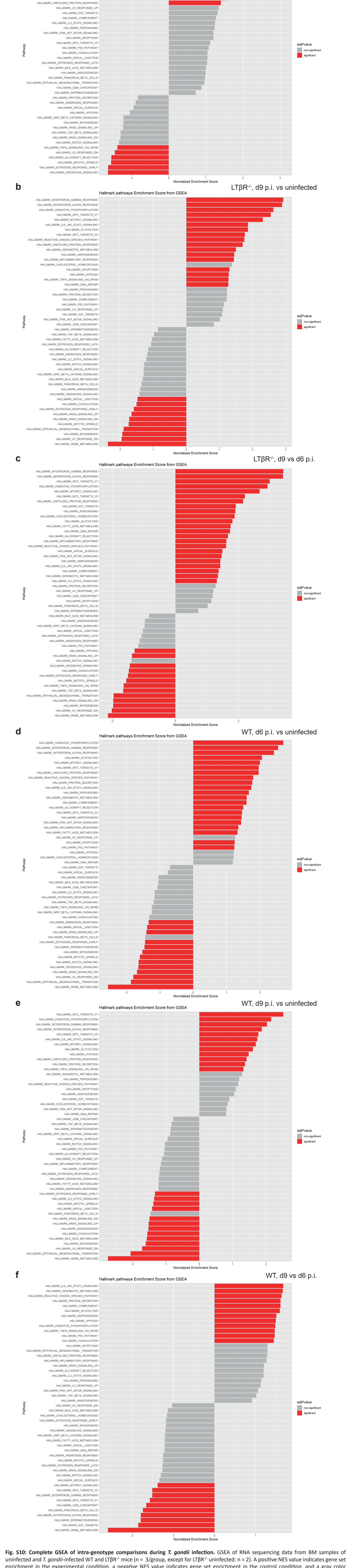

Fig. S10: Complete GSEA of intra-genotype comparisons during *T. gondii* infection. GSEA of RNA sequencing data from BM samples of uninfected and *T. gondii*-infected WT and LTβR$^{-/-}$ mice (n = 3/group, except for LTβR$^{-/-}$ uninfected: n = 2). A positive NES value indicates gene set enrichment in the experimental condition, a negative NES value indicates gene set enrichment in the control condition, and a gray color describes non-significance (adjusted p-value > 0.01). **(a)** LTβR$^{-/-}$ day 6 p.i. and **(b)** LTβR$^{-/-}$ day 9 p.i. vs uninfected controls, displaying enrichment/depletion in infected LTβR$^{-/-}$ BM. **(c)** LTβR$^{-/-}$ day 9 vs day 6 p.i., displaying enrichment/depletion on day 9 p.i. **(d)** WT day 6 p.i. and **(e)** WT day 9 p.i. vs uninfected controls, displaying enrichment/depletion in infected WT BM. **(f)** WT day 9 vs day 6 p.i., displaying enrichment/depletion on day 9 p.i.
