# Supplementary material for: Lymphotoxin beta receptor-/- mice display altered B- and T-cell subpopulations in the bone marrow and peritoneal cavity after Toxoplasma gondii infection
Source: Infect Immun. 2025 Sep 9;93(10):e00408-25. doi: 10.1128/iai.00408-25 (PMC12519803; doi:10.1128/iai.00408-25)
Supplement: Fig. S11 to S13 — Absolute PerC B cell numbers and direct B-1a gating (S11), absolute PerC neutrophils and T cells (S12), and histology of kidney sections from WT and LTßR-/- mice (S13). [file iai.00408-25-s0004.pdf]

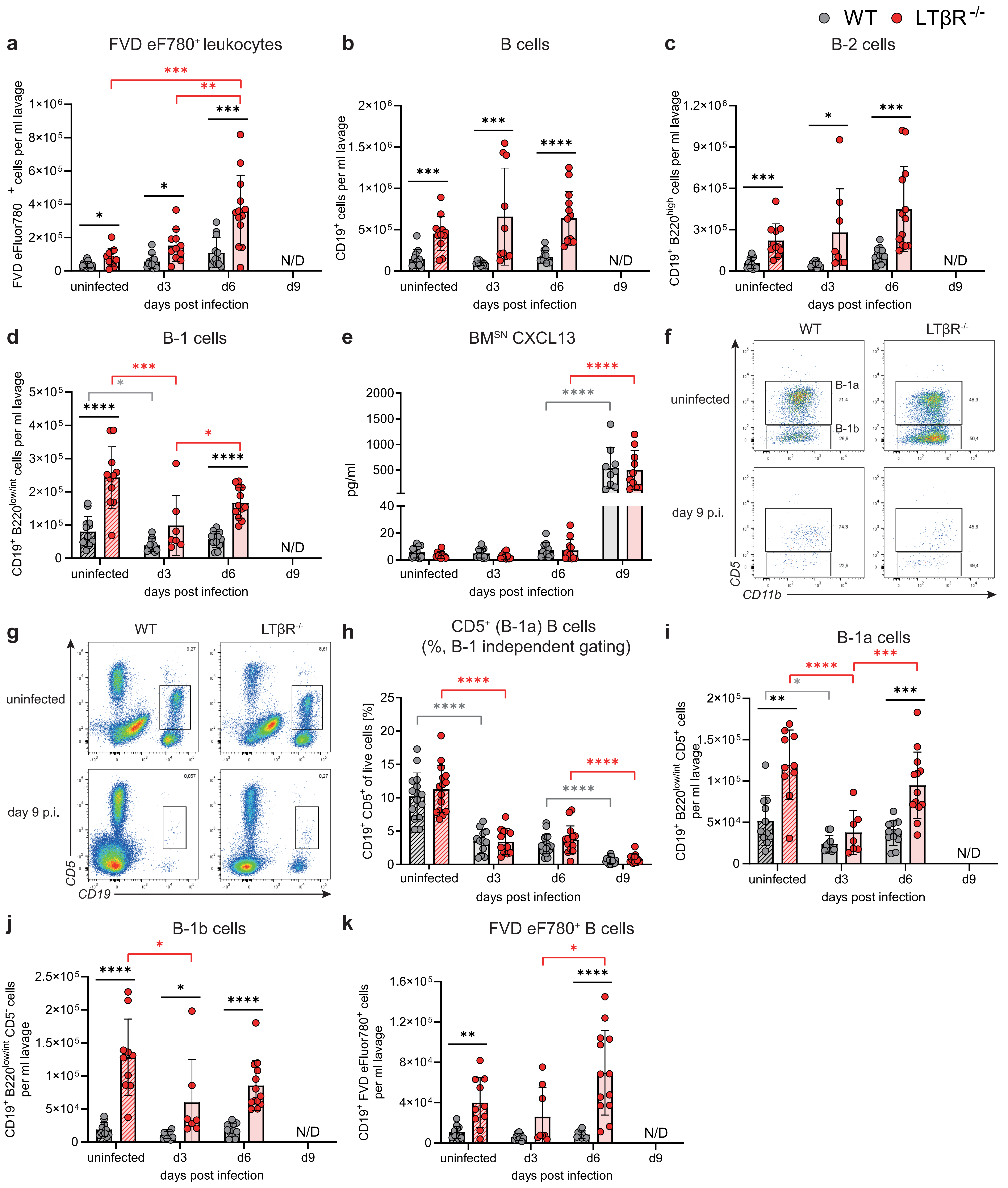

Fig. S11: Absolute numbers of B cell subpopulations and direct B-1a gating in the PerC of *T. gondii*-infected WT and LTβR$^{-/-}$ mice. Using surface marker staining and flow cytometry, the following immune cell populations (for gating strategy see Fig. S8) in the PerC of WT (n ≥ 12/group) and LTβR$^{-/-}$ (n ≥ 10/group) mice were identified: absolute numbers of **(a)** dead leukocytes (FVD eFluor780^{+}), **(b)** B cells (CD19^{+}), **(c)** B-2 cells (CD19^{+} B220^{high}), and **(d)** B-1 cells (CD19^{+} B220$^{low/int}$) per ml peritoneal lavage. **(e)** CXCL13 measured in the BM^{SN} of uninfected and infected WT (n ≥ 9/group) and LTβR-/- (n ≥ 8/group) mice via a bead-based immunoassay (LegendPlex, BioLegend, USA). **(f)** B-1a (CD5^{+}) and B-1b (CD5^{-}) cell gating in a set of representative images. **(g & h)** Alternative, direct detection of B-1a cells (CD19^{+} CD5^{+}) independent of prior B-1 (CD19^{+} B-220$^{low/int}$) cell gating. (g) shows a set of representative images. **(i)** Absolute numbers of B-1a cells (CD19^{+} B220$^{low/int}$ CD5^{+}), **(j)** B-1b cells (CD19^{+} B220$^{low/int}$ CD5^{-}), and **(k)** dead pan-CD19^{+} B cells (CD19^{+} FVD eFluor 780^{+}) per ml peritoneal lavage. No reliable cell counts could be determined for day 9 p.i. due to extensive cell debris and clumping in the peritoneal lavages. While the exclusion of non-single-cell events during flow cytometry allowed for the determination of live cell frequencies, absolute immune cell numbers could not be assessed on day 9 p.i. PerC = peritoneal cavity. BM^{SN} = bone marrow supernatant. All data shown represent at least three independent experiments; symbols represent individual animals and columns represent mean values ± SD. *, P < 0.05; **, P < 0.01; ***, P < 0.001; ****, P < 0.0001. N/D = not determined.

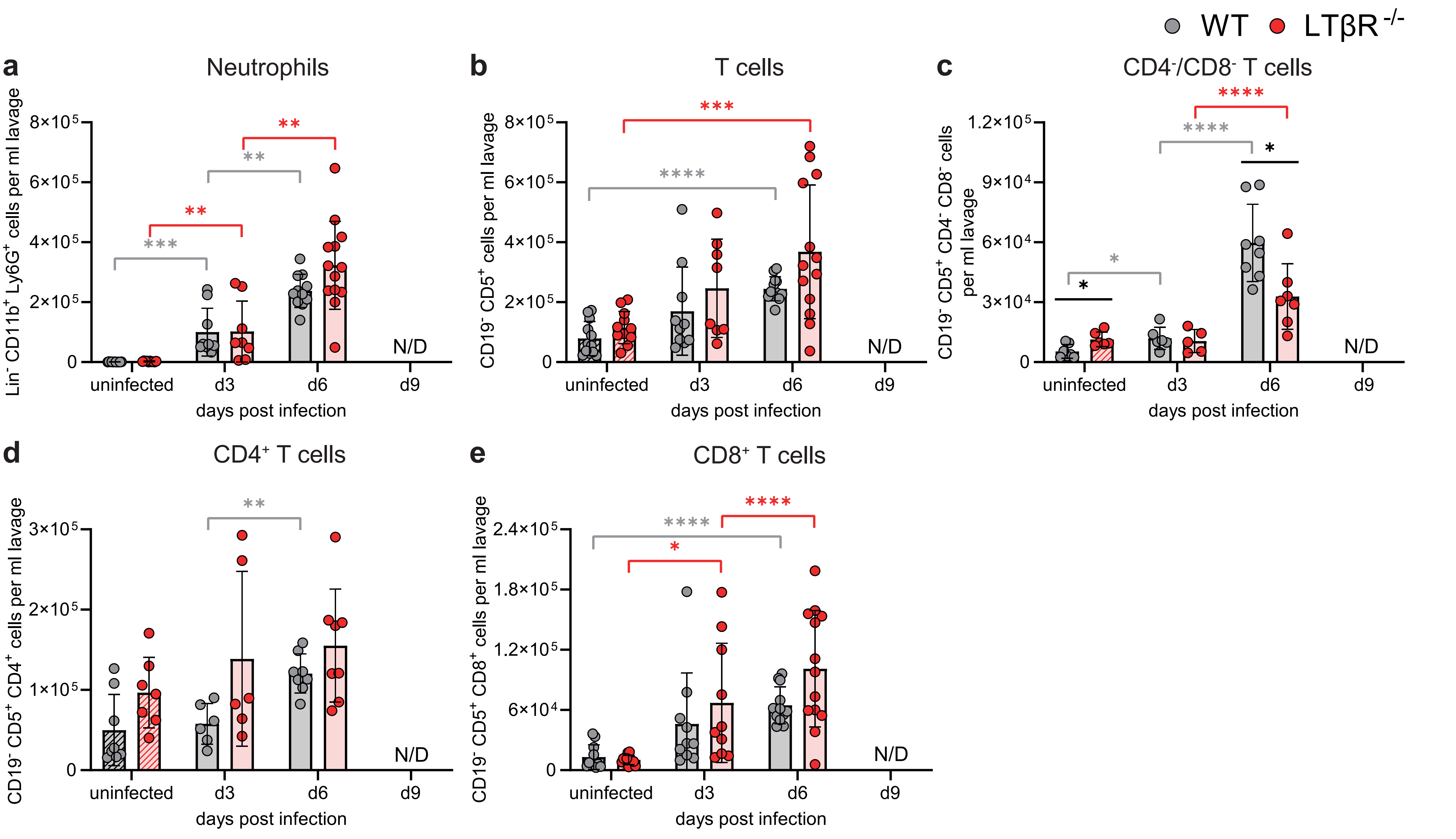

Fig. S12: Absolute numbers of neutrophils and T cell subpopulations in the PerC of *T. gondii*-infected WT and LTβR$^{-/-}$ mice. Using surface marker staining and flow cytometry, the following immune cell populations (for gating strategy see Fig. S8) were identified in the PerC of WT (n ≥ 12/group) and LTβR$^{-/-}$ (n ≥ 10/group) mice: absolute numbers of **(a)** neutrophils (CD19^{-} CD5^{-} NK1.1^{-} CD11b^{+} Ly6G^{+}), **(b)** T cells (CD19^{-} CD5^{+}), **(c)** double-negative T cells (CD19^{-} CD5^{+} CD4^{-} CD8^{-}), **(d)** CD4^{+} T cells (CD19^{-} CD5^{+} CD4^{+}), and **(e)** CD8^{+} T cells (CD19^{-} CD5^{+} CD8^{+}). For (c) and (d): WT: n ≥ 6/group and LTβR$^{-/-}$: n ≥ 5/group. No reliable cell counts could be determined for day 9 p.i. due to extensive cell debris and clumping in the peritoneal lavages. While the exclusion of non-single-cell events during flow cytometry allowed for the determination of live cell frequencies, absolute immune cell numbers could not be assessed on day 9 p.i.. PerC = peritoneal cavity. All data shown represent at least two independent experiments; symbols represent individual animals and columns represent mean values ± SD. *, P < 0.05; **, P < 0.01; ***, P < 0.001; ****, P < 0.0001. N/D = not determined.

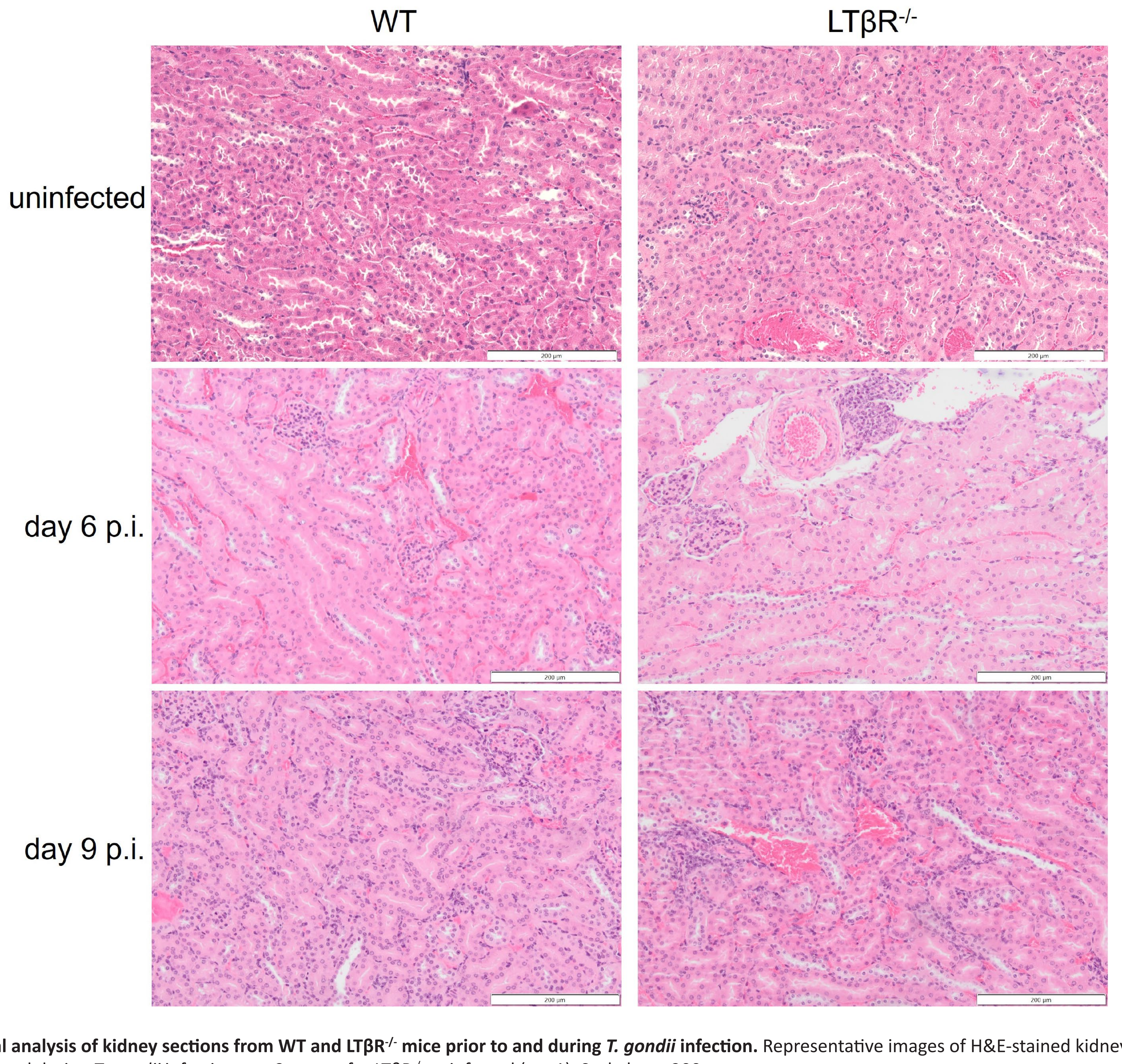

Fig. S13: Histological analysis of kidney sections from WT and LTβR$^{-/-}$ mice prior to and during *T. gondii* infection. Representative images of H&E-stained kidney sections from WT and LTβR$^{-/-}$ mice prior to and during *T. gondii* infection. n = 3 except for LTβR$^{-/-}$ uninfected (n = 1). Scale bars: 200 µm.
